# Automated Flow Peptide Synthesis Enables Engineering of Proteins with Stabilized Transient Binding Pockets

**DOI:** 10.1021/acscentsci.3c01283

**Published:** 2024-02-28

**Authors:** Anna Charalampidou, Thomas Nehls, Christian Meyners, Satish Gandhesiri, Sebastian Pomplun, Bradley L. Pentelute, Frederik Lermyte, Felix Hausch

**Affiliations:** † Clemens-Schöpf-Institute, Department of Chemistry, Technical University of Darmstadt, Peter-Grünberg-Straße 4, 64287 Darmstadt, Germany; ‡ Department of Chemistry, Massachusetts Institute of Technology, 77 Massachusetts Avenue, Cambridge, Massachusetts 02139, United States; § Leiden Academic Centre for Drug Research (LACDR), Leiden University, Einsteinweg 55, 2333 CC Leiden, The Netherlands; ¶ Department of Synthetic Biology, Technical University of Darmstadt, 64287 Darmstadt, Germany

## Abstract

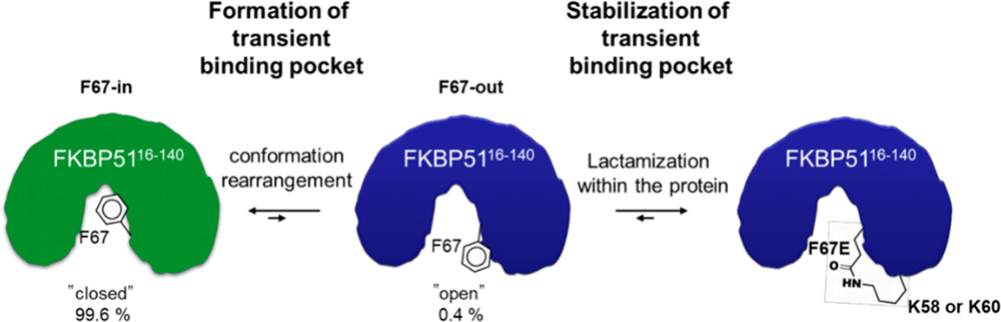

Engineering at the amino acid level is key to enhancing the properties of existing proteins in a desired manner. So far, protein engineering has been dominated by genetic approaches, which have been extremely powerful but only allow for minimal variations beyond the canonical amino acids. Chemical peptide synthesis allows the unrestricted incorporation of a vast set of unnatural amino acids with much broader functionalities, including the incorporation of post-translational modifications or labels. Here we demonstrate the potential of chemical synthesis to generate proteins in a specific conformation, which would have been unattainable by recombinant protein expression. We use recently established rapid automated flow peptide synthesis combined with solid-phase late-stage modifications to rapidly generate a set of FK506-binding protein 51 constructs bearing defined intramolecular lactam bridges. This trapped an otherwise rarely populated transient pocket—as confirmed by crystal structures—which led to an up to 39-fold improved binding affinity for conformation-selective ligands and represents a unique system for the development of ligands for this rare conformation. Overall, our results show how rapid automated flow peptide synthesis can be applied to precision protein engineering.

## Abstract 2

The drug target FKBP51 can be selectively bound in an F67-out conformation, which, however, is populated to only 0.4% in the absence of ligands. By site-specific lactamization within the protein, enabled by automated flow peptide/protein synthesis, the F67-out-like conformation was stabilized.

## Introduction

The identity and special arrangement of amino acids dictate the structure and function of proteins. The ability to modify the side chains of amino acids is key to protein engineering and rational protein design. Molecular evolution-based techniques allow the sampling of large numbers of protein variants and are a powerful approach to engineer proteins with new or optimized function. More recently, computational approaches have matured dramatically to allow the de novo design of new proteins.^1−3^ However, all of these approaches have relied on the variation of the canonical set of genetically encoded amino acids. The properties of proteins can be vastly enhanced by the site-specific incorporation of unnatural amino acids.^4^ This can be biologically achieved by using expression hosts with an expanded genetic code.^5^ However, incorporating two or more different unnatural amino acids is still challenging or even impossible.^6^ Total chemical synthesis, on the other hand, allows for full control of all amino acids. While conventional solid-phase peptide synthesis (SPPS) is limited in length,^7^ methods for dovetailing peptide fragments have been developed and enabled the preparation of full-length proteins.^8,9^ The chemical synthesis of proteins has enabled the site-selective incorporation of defined post-translational modifications and consequently the study of protein variants hardly attainable by recombinant expression.^10−13^ However, peptide ligation approaches are often sequence-dependent and can require cumbersome multistep syntheses, making the preparation of complex protein variants challenging.^14,15^ In contrast, recent advances in rapid automated flow peptide synthesis (AFPS)^16^ allow the rapid chemical synthesis of whole proteins with desired modifications of up to 164 amino acids^17,18^ or even whole protein complexes.^19^ This opens opportunities to engineer designer proteins with unprecedented precision, including proteins with defined conformations.

Small molecule drugs critically rely on suitable surface cavities in their target proteins, so-called binding pockets, to exert a therapeutic effect. These binding pockets are often highly conserved, which makes the development of selective drugs challenging. Historically, structure-based drug discovery has focused on stable, well-defined binding pockets, which—if present—can often be visualized in the apo-states of proteins (i.e., in the absence of ligand). Yet proteins are flexible and can adapt conformations with additional or altered binding pockets. These transient binding pockets can offer significant advantages, such as targeting otherwise intractable targets or enabling otherwise unattainable selectivity.^20,21^ This has been crucial for the development of clinically used or investigated inhibitors for KRas,^22,23^ Abl-BCR,^24−26^ MEK,^27,28^ Mcl-1,^29^ and SHP2.^30^ Transient binding pockets can be identified either by analyzing structural data from protein crystallizations, NMR studies, and cryo-EM experiments, and/or by computational methods.^31−33^ However, the identification and characterization of often sparsely populated conformations in proteins remain a substantial challenge, and most initial hits for such transient binding pockets have been discovered by serendipity so far. Stabilizing transient pockets should allow us to study them in much more detail at the structural and functional level, including focused screening approaches. Rare conformations can be stabilized by genetically engineering the target protein,^34,35^ which, however, is restricted to natural amino acid variations.

**Figure 1:**
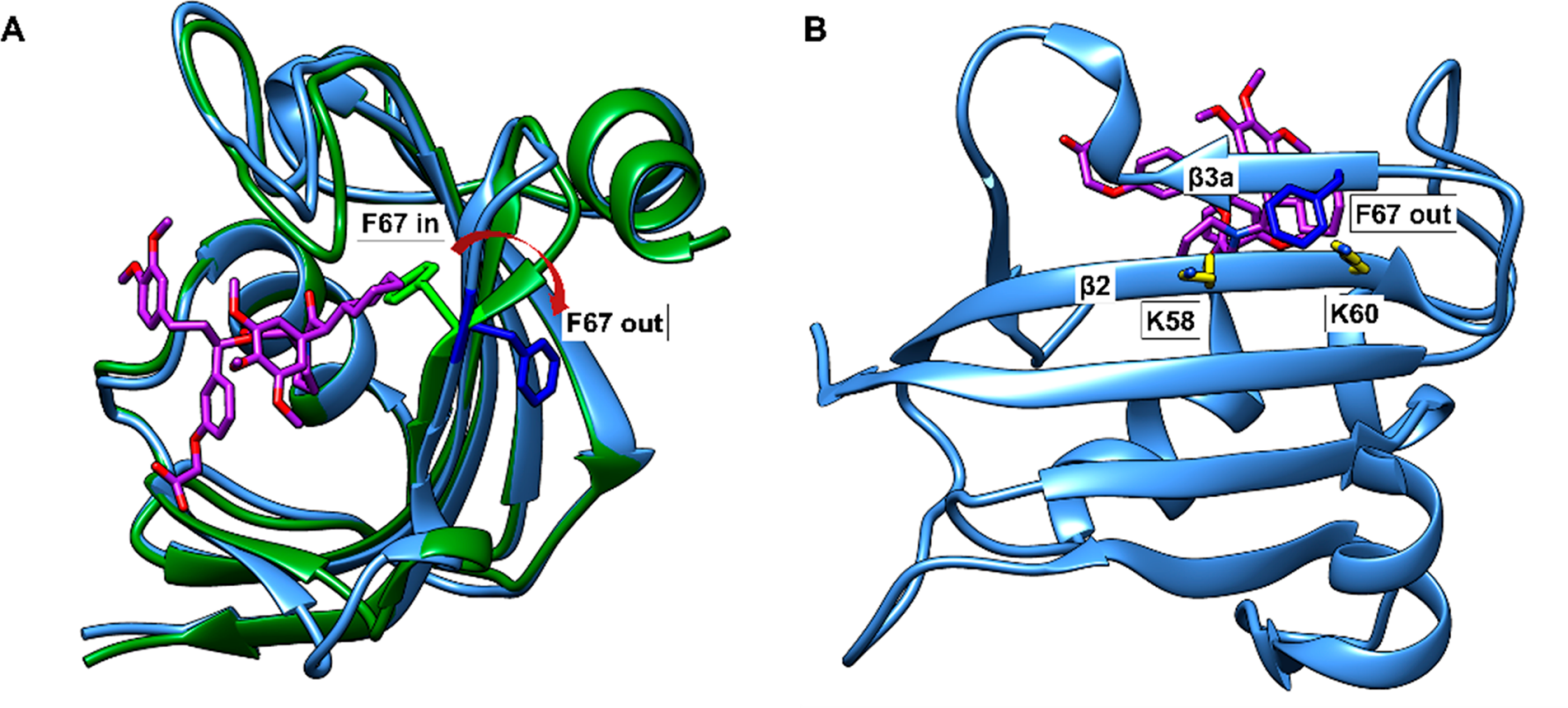
Conformations of FKBP51^16–140^. A: Structural overlay of FKBP51^16–140^ (F67-in) in complex with FK506 (PDB: 3O5R, green cartoon, FK506 omitted for clarity) and FKBP51^16–140^ (F67-out) (PDB: 8CCA, blue cartoon) in complex with SAFit1 (purple sticks). F67 in the critical in- and out-conformations is highlighted as green and blue sticks, respectively. B: View on the backside of the protein binding pocket (PDB: 8CCA). F67 in the β3a-strand and K58 and K60 in the β2-strand are shown as sticks.

Here we used the FK506-binding protein 51 (FKBP51) as a model system to evaluate whether chemically engineered proteins can be trapped in pharmacologically relevant conformations. FKBP51 has emerged as a promising drug target for chronic pain,^36^ obesity,^37,38^ and depression.^39,40^ The major challenge in FKBP51 drug development is selectivity toward its closest homologue FKBP52, as the binding pockets of both proteins are very similar, but the biological functions are opposite. Selectivity for FKBP51 can be achieved by targeting a transient conformation characterized by an outward flip of phenylalanine 67 (F67^in^ and F67^out^, Figure 1A).^41−45^ This conformation is rarely populated in the apo-state of FKBP51,^46^ but enables high selectivity vs FKBP52.^47,48^ To stabilize the transient binding pocket of FKBP51 (F67-out), we aimed to lock this conformation precisely by the formation of an intramolecular amide bond (sites displayed in Figure 1B). We have also explored bis-electrophiles to incorporate intramolecular cross-links between two cysteines at position 67 and 60/58. However, the reaction was prone to side reactions as various side-products were formed and isolation of the desired intramolecularly cross-linked product was not possible. Therefore, we focused on intramolecular amide bond formation by total protein synthesis, which allows a higher degree of reaction control. Intramolecular lactam bridges are well-known to stabilize specific peptide conformations (e.g., α-helices), but this has been put into practice only rarely for intact proteins.^49^

## Results

### Synthesis of Lactam-Bridged FKBP51^16–140^ Variants

**Scheme 1:**
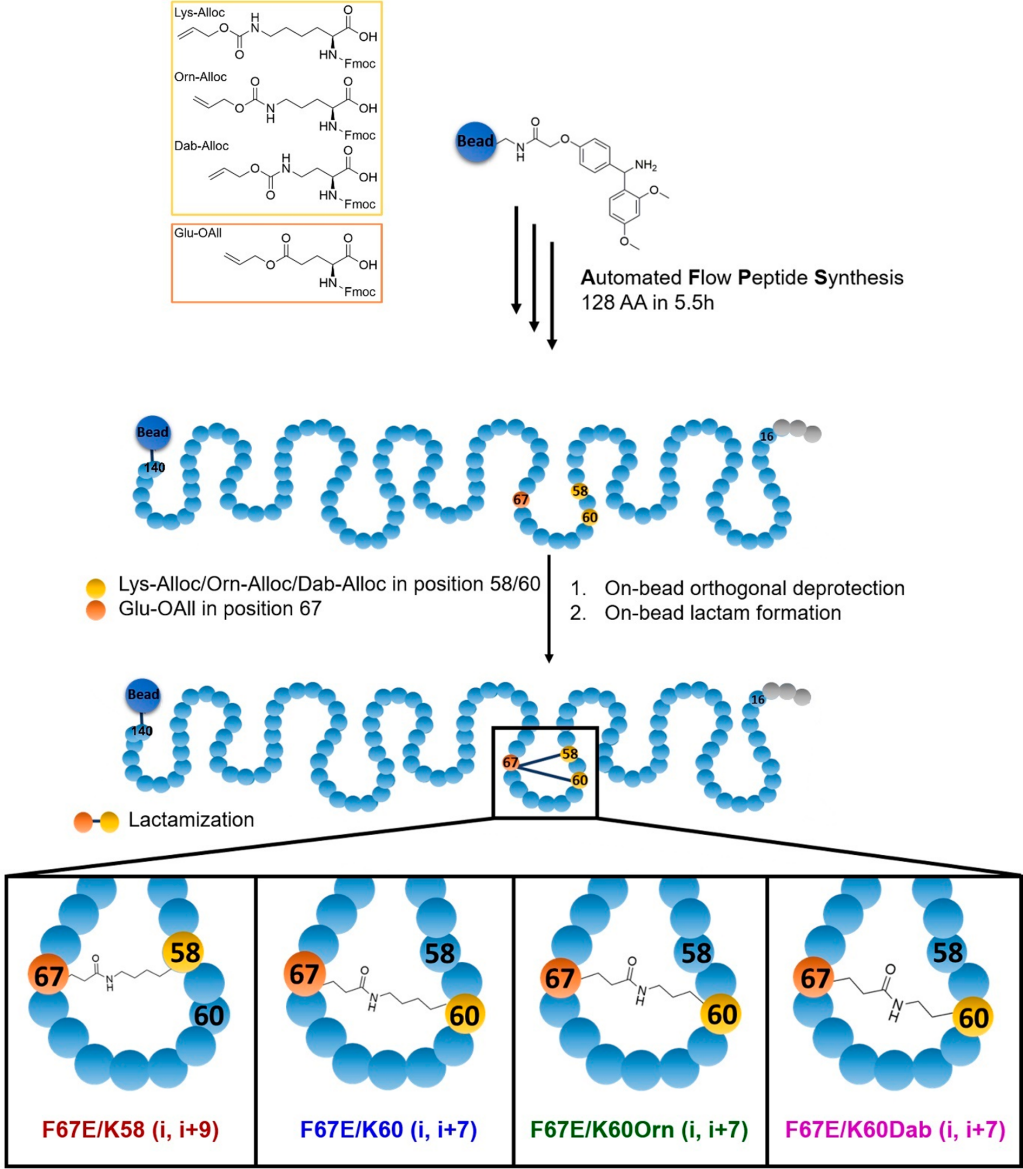
Total Synthesis of Lactam-Bridged FKBP51^16-140^ Variants by Automated Flow Peptide Synthesis (AFPS) AFPS allows the site-specific incorporation of orthogonally protected amino acids, for subsequent on-bead orthogonal deprotection and lactamization. This way, four variants were synthesized with different ring sizes.

To develop FKBP51 variants with a stabilized F67-out-like conformation, AFPS was used to enable site-specific incorporation of unnatural amino acids and building blocks with orthogonal protecting groups. This approach was envisioned to enable the subsequent on-bead orthogonal deprotection and lactam formation, after the coupling of the 128 amino acids of the core FK1 domain of FKBP51 via AFPS. Two positions, K58 and K60, were identified as promising anchor points for lactamization to the residue in position 67 (see Figure 1B). This was expected to trap residue 67 in an out-like conformation and stabilize the β2-β3a-loop by a seven (i, i+7) or nine (i, i+9) amino acid macrocycle. Furthermore, for position 60, the size of the macrocycle was finetuned by incorporating ornithine (Orn) or diaminobutyric acid (Dab) as smaller lysine analogues (see Scheme 1). As a control, the corresponding wild-type-like FKBP51^16–140^ domain was chemically synthesized. For all constructs, the native cysteines (C103A and C107I) and methionines (M48Nle and M97Nle, Nle= Norleucine) were replaced to protect the protein from oxidation. As another control, the recombinantly expressed FKBP51^16–140^ variant was used (see SI for the exact protein sequences).

**Figure 2:**
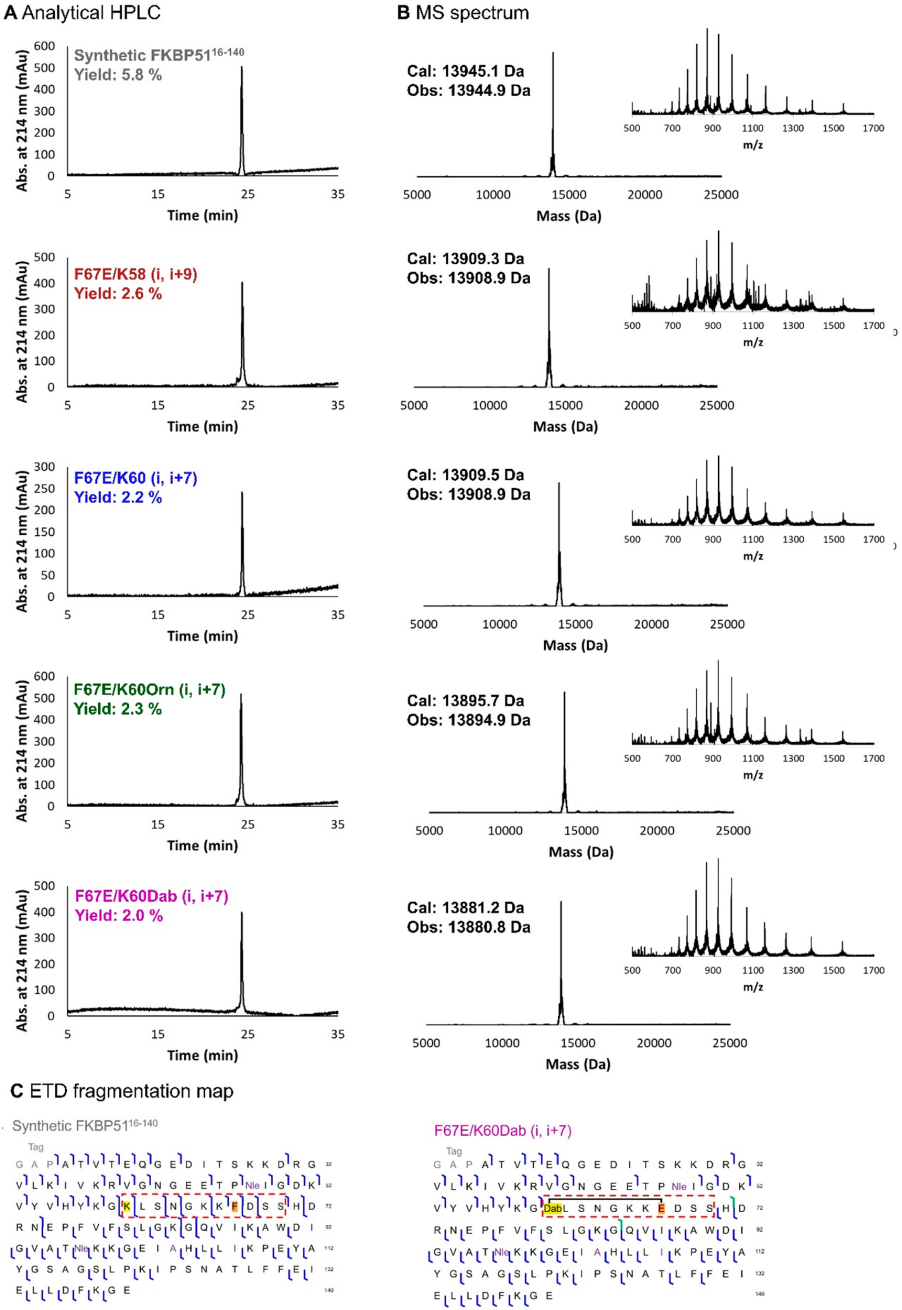
Characterization of chemically synthesized FKBP51^16–140^ variants before refolding and further purification. A: Analytical HPLC chromatograms. B: Deconvoluted MS data. Insets show the MS data prior to deconvolution. C: ETD fragmentation map from top-down mass spectrometry measurements of synthetic and unfolded FKBP51^16–140^ (left) and F67E/K60Dab (i, i+7) (right). Identified c- and z-fragments for ETD are indicated in blue. In green, c-fragments with H_2_O-loss are shown. The fourth residue (alanine) in this construct corresponds to the residue in position 16 in full-length FKBP51, and the numbering in Panel C follows that of the full-length protein. The first three residues (GAP) originate from the purification tag, which were retained in the synthetic constructs for consistency. The purple amino acids indicate the modifications C103A, C107I, M48NLe, and M97Nle. Position 60 is highlighted in yellow, position 67 in orange, and the black line indicates the lactam-bridge formed.

The synthesized variants were purified by preparative reverse phase HPLC and analyzed by analytical HPLC and LC-MS. The variants showed the expected masses and sufficient purity (Figure 2A and B). However, after refolding and additional chromatographic purification, the samples showed a significant improvement in purity when comparing the MS analysis of Figure 2B (data shown in Figure SI 3) and Figure SI 6.

For the synthetic FKBP51^16–140^ variant, we obtained the highest yield of 5.8% and 17.9 mg of lyophilized protein. The cyclized variants were obtained in lower yields, which can be explained by the additional synthesis steps of on-bead orthogonal deprotection and lactam formation.

To confirm the desired lactam formation at the correct site, we chose a top-down mass spectrometry approach using ETD (electron-transfer dissociation) as a fragmentation technique.^50^ In this process, an electron is transferred from a radical anion to the analyte cation, which leads to fragmentation of the backbone amides in proteins. In the synthetic FKBP51^16–140^ variant, we observed large fragments resulting from cleavage between F67 and K58 or K60 (see Figure 2C and Figures SI 4 and SI 5). Similar fragments were observed for the recombinant FKBP51^16–140^. In contrast, for the lactam-bridged variants, fragments resulting from cleavage between F67E/K58, F67E/K60Orn, and F67E/K60Dab are undetectable because the covalent bond formed protects the protein from fragmentation at these sites (see Figure 2C and Figures SI 4 and SI 5). In summary, using top-down ETD, we were able to confirm both the primary sequence and the correct modifications in the synthetic proteins.

### Refolding of Lactam-Bridged FKBP51^16–140^ Variants and Conformational Studies

**Figure 3:**
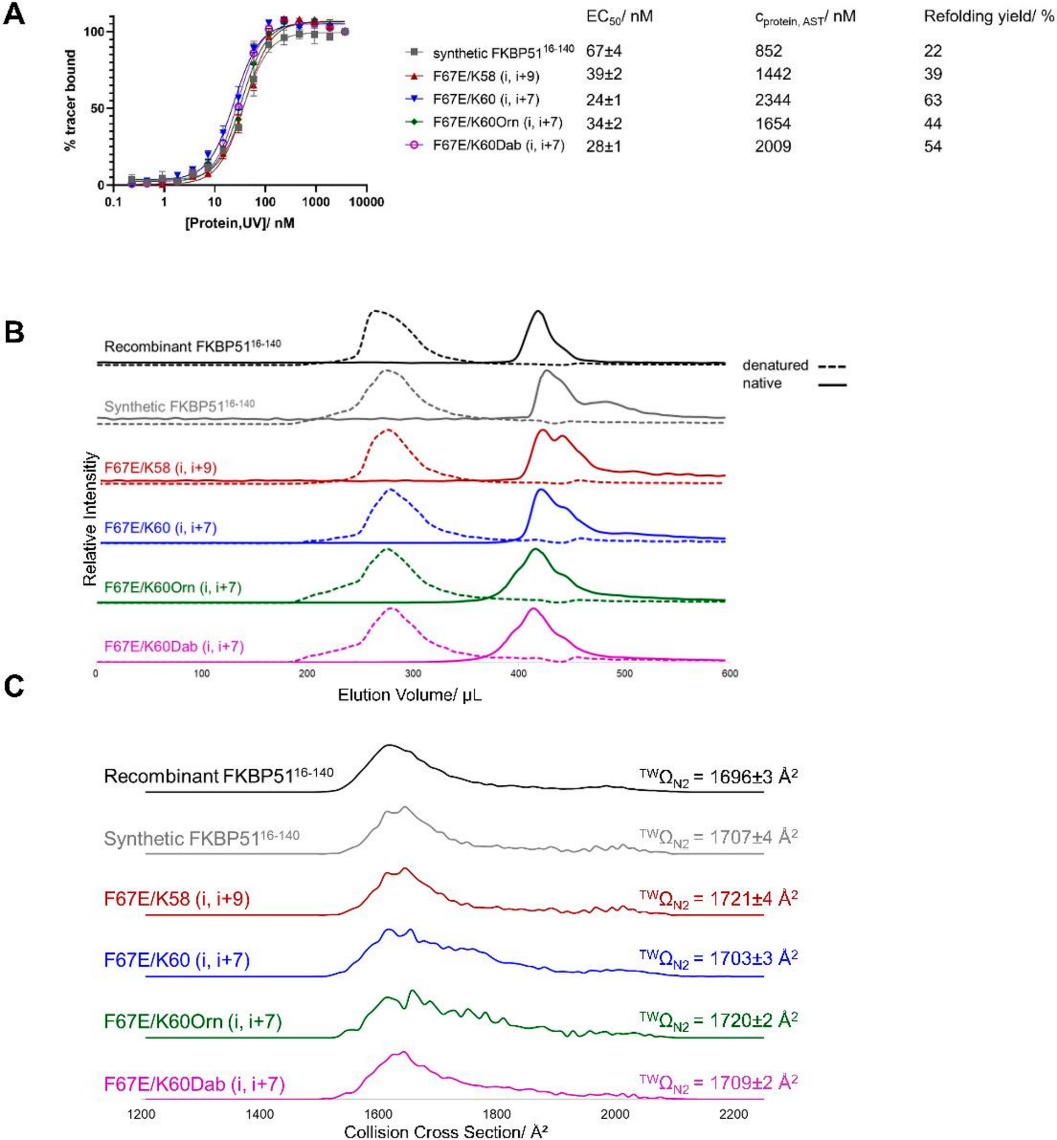
Characterization of the refolded synthetic proteins. A: Active site titration of the synthesized FKBP51^16–140^ variants. The high-affinity tracer SAFit-FL (SAFit1 ligand coupled to fluorescein, final concentration of 30 nM) was used for the active site titration of all five protein variants. Each data point represents the mean of three technical replicates with the corresponding standard error. Protein concentration (determined by UV) range: 230 pM – 3750 nM. B: Size exclusion chromatography–mass spectrometry. Extracted ion chromatograms (native: z = 8+, denatured: z = 16+) of synthetic, lactam-bridged variants in comparison to the synthetic as well as recombinant FKBP51^16–140^ variant. Two conditions were tested: native and denatured. The native runs were conducted with 50 mM ammonium acetate buffer pH 7 (continuous lines) and the denatured runs with 0.2% formic acid in water (dotted lines). C: Arrival time distributions for charge state 8+, measured with traveling wave ion mobility spectrometry with N_2_ as the collision gas. On the right, the collision cross section values are displayed as the top values. Figure SI 5 in the Supporting Information shows the unfolded IMS data for the charge states 8+, 11+, and 18+.

The rapid dilution method was used for refolding the chemically synthesized proteins. Different buffers with different pH values, salt concentrations, and additives were tested, and a refolding buffer with 50 mM Tris-Cl pH 8.5, 9.6 mM NaCl, 0.4 mM KCl, 2 mM MgCl_2_, 2 mM CaCl_2_, 0.5 M arginine, 0.4 M sucrose, and 0.75 M guanidine HCl was found to be optimal. The most important step in refolding was the slow addition of denatured protein (in 6 M guanidine HCl) with stirring to prevent local concentration peaks. The efficiency of refolding was tested by active site titration,^51^ confirming the activity of the refolded proteins and the estimated refolding yields were between 22 to 63% (see Figure 3A).

**Figure 4:**
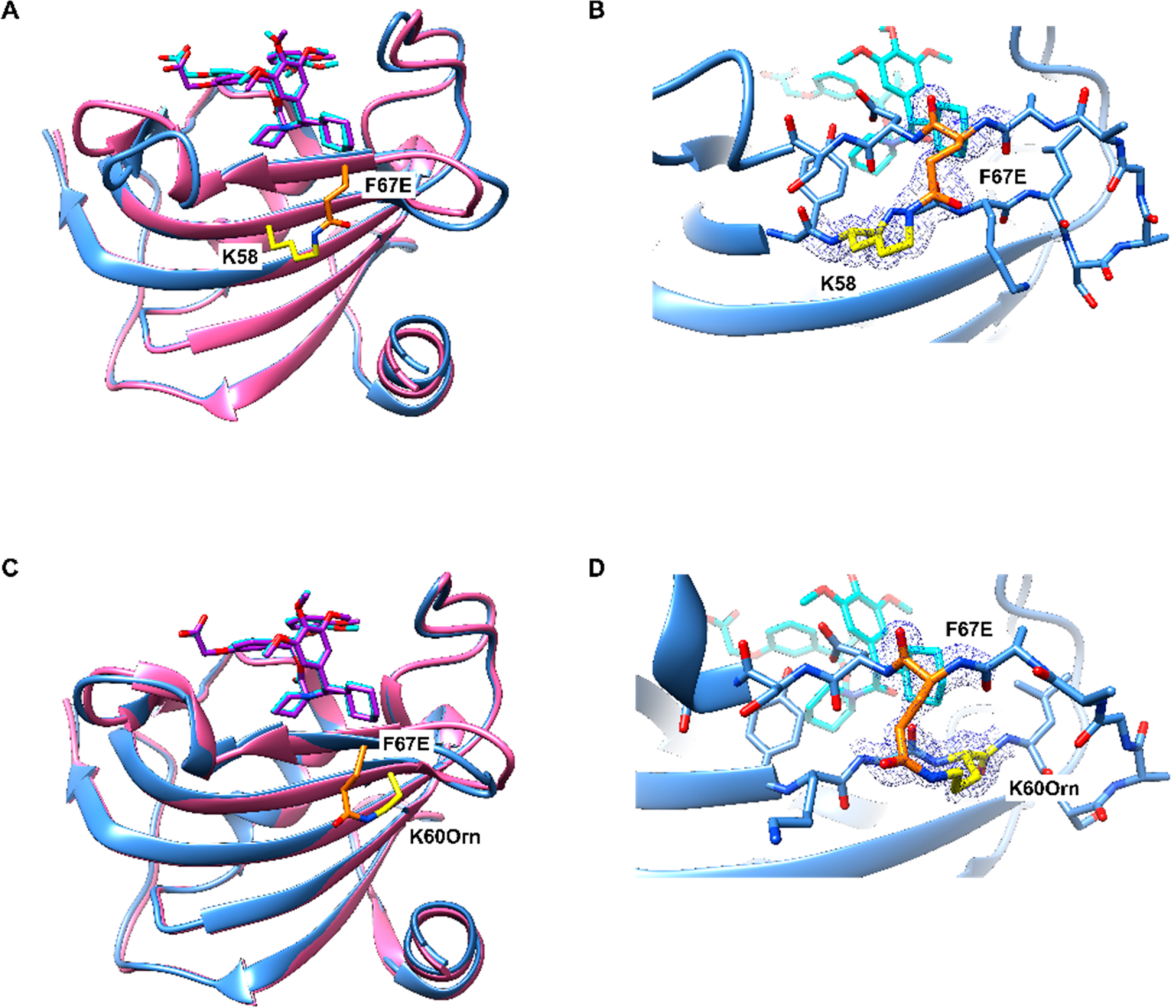
Structures of FKBP51^16–140^, F67E/K58 (i, i+9), and F67E/K60Orn (i, i+7) in complex with the conformation-specific ligand SAFit1. A, C: Stabilized FKBP51^16–140^ F67-out-like conformation depicted as blue cartoons (F67E/K58 (i, i+9) PDB:8PJA, F67E/K60Orn (i, i+7) PDB: 8PJ8) in complex with SAFit1 (cyan sticks) superimposed to wildtype FKBP51^16–140^ (PDB: 8CCA, pink cartoon, SAFit1 shown as purple sticks). B, D: View of the backside of the binding pocket with the electron density maps for the lactam bridge.

For further conformational studies of the refolded synthesized protein variants, size exclusion chromatography–mass spectrometry (SEC-MS) was used. SEC analysis enables us to separate the folded and denatured proteins. All refolded proteins showed significantly longer elution times compared to the denatured proteins, indicative of a more compact conformation, which was similar to the recombinant protein (see Figure 3B). Moreover, for all protein constructs, we observed an average charge distribution of 7.9+ in native SEC-ESI-MS, whereas an average charge distribution of 15.6+ was present in denatured SEC (see SI). Therefore, the proteins appear to have been unfolded in the denaturing environment, making additional basic groups accessible for protonation.^52^ Finally, the proteins were measured under native and denaturing conditions by ion mobility mass spectrometry.^53^ All proteins under native conditions exhibit similar collision cross section (CCS) values, which differed from the denatured proteins (see Figure SI 9). Taken together, the MS results indicate a similar nativelike conformation in solution of the refolded synthesized proteins compared to the recombinantly produced protein. Protein crystallography of the ligand-bound proteins confirmed the desired 3D structure of the synthesized and refolded proteins FKBP51^16–140^ F67E/K58 (i, i+9) and F67E/K60Orn (i, i+7) (Figure 4A and C). The protein fold, the ligand binding mode, and the conformation of the β3-strand were identical to recombinant FKBP51. Notably, the side chains of F67E/K58 and F67K60 were well resolved, which unambiguously confirmed the desired lactam bridge and its F67-out mimicking conformation (see Figure 4B and D).

### Ligand Binding of Lactam-Bridged FKBP51^16–140^ Variants

**Figure 5:**
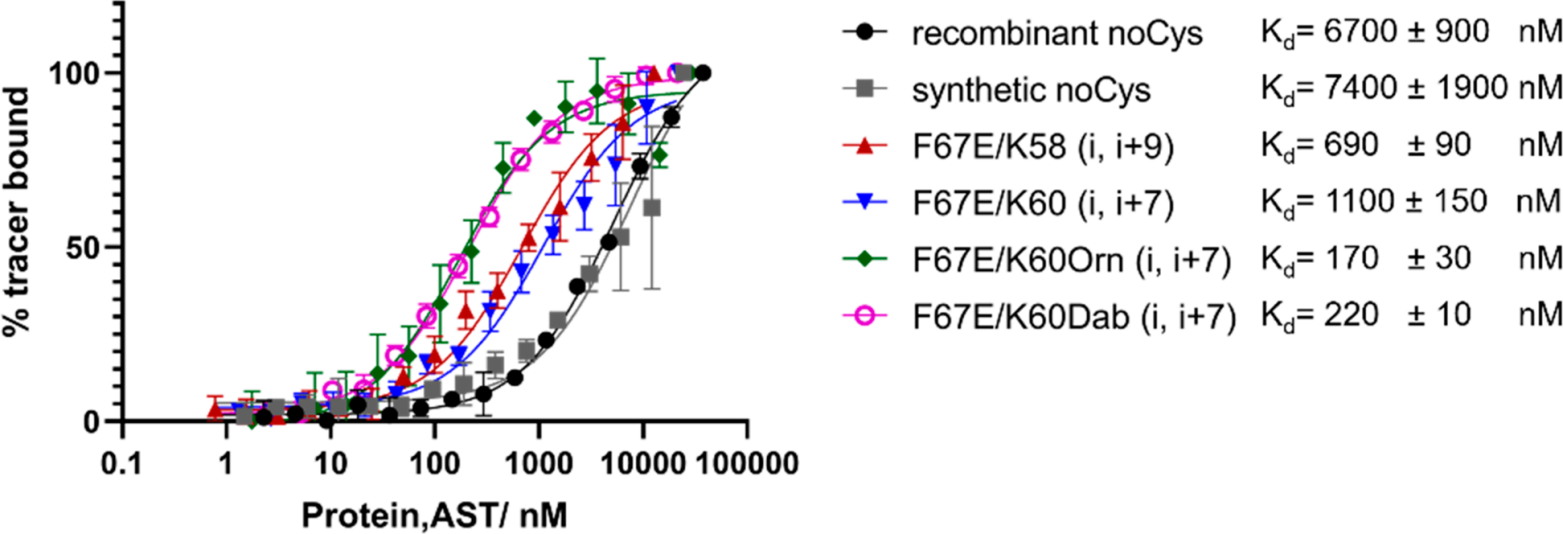
FP-Assay with a low affinity binding tracer. Binding curves of the synthesized (gray curve) and cyclized FKBP51^16–140^ variants (blue, red, green and magenta curves) and, as a control, the recombinant FKBP51^16–140^ protein (black curve) with a low affinity tracer (low affinity SAFit1 based fluorescein tracer, 1 nM). Each data point is indicated as the mean of three technical replicates with the respective standard errors.

Lactamization within the protein was expected to shift the conformational equilibrium toward the F67-out-like conformation. This should result in a higher binding affinity of conformation-specific ligands, as the energetic penalty to adopt the F67-out-like conformation is reduced. Indeed, when we compare the *K*_d_ values of recombinant FKBP51^16–140^ with the synthesized and cyclized variants, we observe a 6–10-fold tighter binding for the lysine/glutamic acid cyclized variants. When lysine is replaced by the smaller ornithine, up to 39-fold improvement is achieved (Figure 5).

## Discussion

Here, we report how precision protein engineering enables stabilization of a transient binding pocket, resulting in improved affinity toward conformation-specific ligands. Our model protein FKBP51 can adopt a selectivity-enabling conformation,^41^ but at a large energetic penalty (approximately 14 kJ/mol^46^). Like for many transient pockets, this precludes most biophysical approaches from directly studying the desired conformation. More importantly, it poses a substantial additional hurdle for the discovery of initial hits for this conformation since these immediately must pay the costs associated with the conformational rearrangements. Here we show that locking the critical amino acid at position 67 in an outward conformation results in a 39-fold improved binding affinity for conformation-specific ligands. This corresponds to an enhanced binding affinity of approximately 9 kJ/mol that was prepaid by the macrocyclization approach.

Different approaches can be envisioned for conformationally selective protein stabilization. Classical protein engineering requires sophisticated conformational read-outs and is restricted to the variation of natural amino acids.^35^ More tailored approaches require the incorporation of unnatural amino acids. This can be achieved by amber codon suppression technology.^5^ However, the simultaneous incorporation of two different unnatural amino acids with orthogonal reactivity remains a substantial challenge. Total chemical protein synthesis on the other hand allows full control of all available amino acid analogues at each position, thus tapping into the vast possibilities of modern peptide chemistry. Bacchi et al. demonstrated for FKBP12.6 that chemical protein synthesis by native chemical ligation was suitable to obtain the 107-amino-acid-long protein. The protein in its wild-type sequence was also successfully refolded and showed catalytic activity.^54^ Here, we used rapid automated flow peptide synthesis, which allowed the one-shot synthesis of the 128-amino-acid-long protein and incorporation of amino acids with orthogonal protecting groups (Alloc and O-Allyl) for on-bead lactam formation within the protein. Our results show how total protein synthesis can enhance our capabilities for protein engineering, allowing the modulation of protein active sites with unprecedented precision.

## Supporting Information Available

Experimental procedures and ETD data of all variants and IMS data under denaturing conditions (PDF)
Transparent Peer Review report available (PDF)

## ABBREVIATIONS

FKBP51: FK506 binding protein 51
AFPS: automated flow peptide synthesis
IMS: ion mobility mass spectrometry
ETD: electron-transfer dissociation
CCS: collision cross section
