# Supplementary material for: Automated Flow Peptide Synthesis Enables Engineering of Proteins with Stabilized Transient Binding Pockets
Source: ACS Cent Sci. 2024 Feb 28;10(3):649–57. doi: 10.1021/acscentsci.3c01283 (PMC10979424; doi:10.1021/acscentsci.3c01283)
Supplement: Supplementary file 1 — oc3c01283_si_001.pdf [file oc3c01283_si_001.pdf]

Supplementary Materials for

Automated flow peptide synthesis enables engineering of proteins with stabilized transient binding pockets

Anna Charalampidou[a,+], Thomas Nehls[a,+], Christian Meyners[a], Satish Gandhesiri[b], Sebastian Pomplun[c], Bradley L. Pentelute[b], Frederik Lermyte[a,d], Felix Hausch[a,d]

+ both authors contributed equally to this work

Anna Charalampidou – Clemens-Schöpf-Institute, Department of Chemistry, Technical University of Darmstadt, Peter-Grünberg-Straße 4, 64287 Darmstadt, Germany, email: anna.charalampidou@tu-darmstadt.de

Thomas Nehls – Clemens-Schöpf-Institute, Department of Chemistry, Technical University of Darmstadt, Peter-Grünberg-Straße 4, 64287 Darmstadt, Germany, email: Thomas.nehls@tu-darmstadt.de

Christian Meyners– Clemens-Schöpf-Institute, Department of Chemistry, Technical University of Darmstadt, Peter-Grünberg-Straße 4, 64287 Darmstadt, Germany, email: Christian_stephan.meyners@tu-darmstadt.de

Satish Gandhesiri – Department of Chemistry, Massachusetts Institute of Technology, 77 Massachusetts Avenue, Cam-bridge, MA 02139, USA, email: satishg@mit.edu

Sebastian Pomplun – Leiden Academic Centre for Drug Research (LACDR), Leiden University, Einsteinweg 55, 2333 CC Leiden, The Netherlands, email: s.j.pomplun@lacdr.leidenuniv.nl

Bradley L. Pentelute – Department of Chemistry, Massachusetts Institute of Technology, 77 Massachusetts Avenue, Cam-bridge, MA 02139, USA, email: blp@mit.edu

Frederik Lermyte – Clemens-Schöpf-Institute, Department of Chemistry, Technical University of Darmstadt, Peter-Grünberg-Straße 4, 64287 Darmstadt, Germany; Centre for Synthetic Biology, Technical University of Darmstadt, Darmstadt, Germany, email: frederik.lermyte@tu-darmstadt.de

This PDF file includes:

25 pages, 9 SI figures, 2 SI tables

Material and Methods

Additional Data: Analytical data, ETD fragmentation maps, MS data of SEC-MS measurements, MS data of CCS measurements, CCS data for denatured samples

Materials and Methods S3
Material S3
Synthesis of FKBP51 FK1 variants by AFPS S3
Automated flow peptide synthesis S3
Orthogonal on-bead deprotection of Alloc and OAll groups S5
Lactamization S5
Cleavage S5
Preparative mass-directed RP-HPLC S6
Characterization of the FKBP51 FK1 variants S6
LC-MS S6
Analytical HPLC S6
ETD measurements S6
Refolding of the synthesized FKBP51 variants S7
Conformation-sensitive MS S8
Size exclusion chromatography – native & denatured S8
Ion mobility mass spectrometry– native & denatured S8
Protein Crystallization S10
Protein production and purification S12
Fluorescence polarization S13
Synthesis of low affinity tracer (4-((3-(4-((((S)-1-((S)-2-Cyclohexyl-2-(3,4,5-trimethoxyphenyl)acetyl)piperidine-2-carbonyl)oxy)methyl)-1H-1,2,3-triazol-1-yl)propyl)carbamoyl)-2-(6-hydroxy-3-oxo-3H-xanthen-9-yl)benzoic acid) S15
Additional Data S16
References S23

Materials and Methods

Safety Statement: No unexpected or unusually high safety hazards were encountered.

Material

All reagents were used as received. The Fmoc-protected amino acids were purchased from CreoSalus or Novabiochem or in case of Fmoc-His(Boc)-OH from ChemPrep. All original methionine residues in the sequences were exchanged with Norleucine (Fmoc-NLeu-OH) which was purchased from ChemImpex. The orthogonal protected lysin (Fmoc-Lys(Alloc)-OH) and its derivatives (Fmoc-Orn(Alloc)-OH and Fmoc-Dab(Alloc)-OH) as well as the orthogonally protected glumatic acid (Fmoc-Glu(OAll)-OH) were purchased from ChemImpex. As solid support in AFPS H-Rink amide ChemMatrix LL was used (PCAS BioMatrix, Loading: 0.40-0.60 mmol/g and bead size: 100-200 mesh dry). The activators HATU (*O*-(7-azabenzotriazol-1-yl)-*N,N,N′,N′*-tetramethyluronium hexafluorophosphate) and PyAOP (7-azabenzotriazol-1-yloxy)tripyrrolidinophosphonium hexafluorophosphate) are from ChemImpex. DCM (dichlormethane) and DMF (*N*,*N*-diisopropylethylamine) as solvents were purchased from EMD Millipore and each DMF bottle contained an AldraAmine trapping packet from Sigma-Aldrich. DIPEA (diisopropylethylamine), piperidine, TFA (trifluoroacetic acid), FA (formic acid), DMSO (dimethyl sulfoxide), $Pd(PPh_3)_4$ (tetrakis(triphenylphosphine)palladium(0)), $PhSiH_3$ (phenyl silane), EDT (1,2- ethanedithiol), thioanisole, phenol, acetonitrile (HPLC grade), were purchased from Sigma-Aldrich. Water was deionized by a Milli-Q Reference water purification system from Millipore. The acetonitrile (LC-MS) and formic acid (p.a.) for the SEC-MS, denatured CCS, and ETD measurements were from Merck. Holomyoglobin (equine), ubiquitin (bovine) and cytochrome c (equine) for CCS calibration, as well as 1,4-dicyanobenzene (ETD reagent) were from Merck. The water (LC-MS) for SEC-MS, CCS and ETD measurements was from Fisher Scientific. For the 200 mM ammonium acetate solution we diluted 7.5 M ammonium acetate stock solution from Merck with water (LC-MS) from Fisher Scientific. We calibrated the Synapt XS and Synapt G2-S with sodium iodide solution from Waters.

Synthesis of FKBP51 FK1 variants by AFPS

Five FKBP51$^{16\text{-}140}$ variants were synthesized by Automated Flow Peptide Synthesis in the Pentelute Group according to the established conditions described in (*1, 2*).

Automated flow peptide synthesis

Approximately 130 mg of H-Rink amide ChemMatrix resin (loading 0.18 mmol/g) was loaded into a fritted syringe (6 mL), swollen in amine-free DMF for 15 min and washed alternatingly with DCM and DMF. The beads were loaded into the reactor of the in the Pentelute Lab, self-build automated flow peptide synthesizer (“Peptidator”) (*2*). The general flow rate was 40 mL/min, the temperature in the loop was 90 °C and 85-90 °C in the reactor. The first step

was a 60 s wash step at elevated temperatures with 40 mL of amine-free DMF. The coupling-deprotection cycle was repeated for all additional monomers used in the sequences.

The shown sequence represents the recombinantly FKBP51$^{16\text{-}140}$ sequence:

tag 16 21 31 41 51 61
GAPATVTE QGEDITSKKD RGVLKIVKRV GNGEETPMIG DKVYVHYKGK LSNGKKFDSS
81 91 101 111 121
HDRNEPFVFS LGKGQVIKAW DIGVATMKKG EIAHLLIKPE YAYGSAGSLP KIPSNATLFF
140
EIELLDFKGE

The following alterations compared to the wildtype were made:

- N-terminus starts at position A16
- The first three amino acids are Tag remains
- A19T mutation for crystallography (*3*)
- Cysteines are abolished: C103A and C107I
- M48 and M97 are replaced by Norleucin

For the synthetic variants the presented sequence was used as template. Additional modification specific to the variants are summarized in the table below.

Table SI1: Alterations in protein sequence specific to the variants.

**Variant**	**Sequence Alterations**
F67E/K58 (i, i+9)	- F67 is replaced by Fmoc-Glu(OAll)-OH<br>- For K58 the building block Fmoc-Lys(Alloc)-OH is used<br>- The N-terminus is protected by incorporating Boc-Gly-OH
F67E/K60 (i, i+7)	- F67 is replaced by Fmoc-Glu(OAll)-OH<br>- For K60 the building block Fmoc-Lys(Alloc)-OH is used<br>- The N-terminus is protected by incorporating Boc-Gly-OH

F67E/K60Orn (i, i+7)	- F67 is replaced by Fmoc-Glu(OAll)-OH<br>- K60 is replaced by the building block Fmoc-Orn(Alloc)-OH<br>- The N-terminus is protected by incorporating Boc-Gly-OH
F67E/K60Dab (i, i+7)	- F67 is replaced by Fmoc-Glu(OAll)-OH<br>- K60 is replaced by the building block Fmoc-Dab(Alloc)-OH<br>- The N-terminus is protected by incorporating Boc-Gly-OH

Orthogonal on-bead deprotection of Alloc and OAll groups

After synthesis, the beads (loading: 0.0221 mmol, 1 eq.) were washed thoroughly with amine free DMF (3 x 5 mL) and DCM (3 x 5 mL) before drying them in a vacuum chamber. 5.1 mg $Pd(PPh_3)_4$ (0.00442 mmol, 0.2 eq.) was dissolved in 4 mL DCM and 54 µL of $PhSiH_3$ (0.442 mmol, 20 eq.) was added. The mixture was added to the syringe containing the dried beads. The suspension was incubated for 30 min at room temperature. This step was performed twice. The beads were then washed with DCM (3 x 5 mL) and subsequently DMF (3 x 5 mL).

Lactamization

The beads (loading: 0.0221 mmol, 1 eq.) were mixed with a solution of 57.6 mg PyAOP (0.1105 mmol, 5 eq.) and 80 µL DIPEA (0.442 mmol, 20 eq.) in 2 mL amine free DMF. The reaction mixture was incubated for 4 h at room temperature on a rolling device. The beads were washed thoroughly with DMF for 3 x 5 min and subsequently with DCM for 3 x 5 min. The beads were then dried in a vacuum chamber.

Cleavage

The dried beads were mixed with cleavage solution to remove the remaining protecting groups and release the proteins from the beads. For approximately 200 mg of beads, 5 mL of cleavage solution (82.5 % TFA, 5 % water, 5 % phenol, 5 % thioanisole, 2.5 % EDT) was added and kept on a rolling device for 3 h at room temperature. The proteins were precipitated in 40-45 mL ice cold diethyl ether and the precipitate was collected by centrifugation while the supernatant was discarded. Traces of diethyl ether and TFA were removed as much as possible by evaporation with a nitrogen gas flow. The proteins were dissolved in 50 % acetonitrile (0.1% TFA) in water and dried by lyophilization.

Preparative mass-directed RP-HPLC

30-40 mg of the lyophilized crude proteins were dissolved in 5 mL buffer containing 6 M guanidine hydrochloride and 100 mM Tris at pH 8. All samples were filtrated with a nylon 0.22 µm syringe filter before loading to the column. For all HPLC purifications, a gradient of 28 % to 48 % solvent B in 100 min was used. Solvent A was MQ water with 0.1 % TFA and Solvent B was HPLC-grade acetonitrile with 0.1 % TFA. The semipreparative Agilent Zorbax 300SB-C3 column (9.4 mm × 250 mm, 5-µm particle size) column was used at a temperature of 60 °C and with a flow rate of 4 mL/min. The pure fractions are combined, lyophilized, weighted and used for further characterization.

Characterization of the FKBP51 FK1 variants

LC-MS

To check the mass of the synthesized proteins, a 1 mg/mL solution was prepared and diluted with 50 % HPLC acetonitrile in water with 0.1% TFA as additive to a 0.1 mg/mL solution. The solutions were filtered and measured on an Agilent 6545 Accurate-Mass Q-TOF LCMS system. The used solvent composition is for solvent A MQ water with 0.1% formic acid as additive and for solvent B HPLC grade acetonitrile with 0.1% formic acid. Method used: C3-1-95 in 15 min using the Agilent Zorbax 300SB-C3 column (2.1 mm × 150 mm, 5-µm particle size).

Analytical HPLC

The purity of the samples was analyzed by analytical HPLC. The measurements were performed on Agilent Technologies 1200 Series instrument with a Kinetex® 2.6 µm (C18, 100 Å, 100 x 2.1 mm) column, with solvent A being MQ water and solvent B being HPLC-grade acetonitrile with 0.1 % TFA as additive for each solvent. The flow rate was 0.375 mL/min and a linear gradient of 5-65 % solvent B in 60 min was used for all samples. For sample preparation a 1 mg/mL protein solution in 50 % solvent B in solvent A was prepared and filtered. 5 µL of each sample was injected.

ETD measurements

For top-down electron transfer dissociation (ETD) measurements, the lyophilized proteins were dissolved in 200 mM ammonium acetate (pH 7, LC-MS grade) at a final concentration of 30 µM. To remove small molecules from the synthesis, samples were buffer exchanged 5-times with 200 mM ammonium acetate buffer (pH 7, LC-MS quality) at 14,000 x g for 30 min using

Merck 3k Amicon® Ultra centrifugal filters. At the end, 50 µL protein solution in 200 mM ammonium acetate buffer was obtained and mixed with 22 µL acetonitrile, 0.3 % formic acid, giving a ratio of 70:30 H_2O:ACN and about 0.1 % formic acid. The samples were measured with direct infusion nanoESI on a Synapt XS from Waters. 10 µL protein solution was loaded in a self-pulled nanoESI capillary (Micropipette Puller Model P-97 from Sutter Instrument). Settings were as follows: Capillary Voltage between 1.0 and 1.5 kV, Sampling Cone 50 V, Source Offset 20 V, Source Temperature 100 °C, Cone Gas Flow 200 L/h, and Purge Gas Flow 300 mL/h for the nanoESI source. The ETD reagent used was 1,4-dicyanobenzene with a Make-Up gas flow of 50 mL/min, flowing through the Glow Discharge at a current of 55 µA and a voltage of 0.9 kV. In the process, the trap cell was filled with anion radicals at an interval of one second for a duration of 0.1 seconds. The pressure inside the helium filled trap cell was around $5.5 \cdot 10^{-2}$ mbar with a trap RF of 450 kHz. The transfer cell was filled with argon at a pressure of around $4.0 \cdot 10^{-2}$ mbar and a collision energy of 5 V was used. The MS/MS experiment was performed to isolate a charge state using a quadrupole equipped with a 300 kHz RF generator and set to an LM resolution of 14 and a HM resolution of 16. The TOF was set in resolution mode with a mass range of 50 to 2000 m/z, and measurements were performed for at least 20 minutes. Before the measurements, the instrument was calibrated with sodium iodide up to 5000 m/z.

Internal recalibration was performed based on the fragments c_4 (z = 1, 314.18232 m/z), z_7 (z = 1, 691.3541 m/z), c_8 (z = 1, 744.38869 m/z), z_{16} (z = 2, 934.9819 m/z), c_{19} (z = 2, 1001.5023 m/z), c_{23} (z = 2, 1200.14837 m/z) and z_{30} (z = 1, 1630.8362 m/z), if they were present. The analysis was performed in two ways: manually and software-assisted. For the manual evaluation the spectra were accumulated, smoothed with Savitzky-Golay-filter with a smooth-window of 3 and a 2 times repetition, and centered with the median. The observed masses were compared with the theoretical mass list of possible fragments, which were calculated with Proteomics Toolkit (*4*). The second evaluation was done with the software MASH Native (*5*). Therefore, the UniDec algorithm (*6*) accumulates the spectra and then fits them with eTRASH (*7*) with a peak background ratio of 2, S/N Threshold of 2, maximum charge state of 17, mass range 100 to 2000 m/z, and a maximum mass of 13950 Da. In addition, we made sure that at least 4 isotopes of a fragment were present for it to be identified as a fragment. We looked for c- and z-fragments, as these are the typical fragments generated in ETD (*8, 9*).

Refolding of the synthesized FKBP51 variants

1 mg of the synthetic protein was dissolved in 250 µL denaturing buffer (6 M guanidine HCl, 100 mM Tris pH 8) and incubated at room temperature for 15 min. In 25 wells of a 96-well plate 10 µL of the protein solution was added slowly to 190 µL of Refolding Buffer (50 mM Tris-Cl pH 8.5, 9.6 mM NaCl, 0.4 mM KCl, 2 mM $MgCl_2$, 2 mM $CaCl_2$, 0.5 M arginine, 0.4 M sucrose,

0.75 M guanidine HCl) under stirring. The plate was incubated for 1 h at 4°C before combining the fractions and exchanging the buffer to 20 mM HEPES pH 8, 20 mM NaCl and concentrating the samples using Amicon® Ultra 2 mL centrifugal filters (MWCO 10kDa).

Conformation-sensitive MS

Size exclusion chromatography – native & denatured

The refolded proteins and recombinant protein stock solutions were buffer-exchanged four times in 200 mM ammonium acetate buffer (pH 7, LC-MS grade) at 14,000 x g for 30 min using 3k Amicon® Ultra centrifugal filters (Merck). The buffer-exchanged protein solutions were diluted with 200 mM ammonium acetate buffer to an initial concentration of 10 µM. For the denatured measurements the protein solutions were diluted to a final protein concentration of 1 µM with 0.2 % formic acid in 30:70 acetonitrile:water (LC-MS grade).

The SEC-MS measurements were performed with an HDX setup from Waters. This included a PAL RTC Autosampler from Leap, a UHPLC with µBinary Pump and Auxiliary Pump, the HDX Manager and a Synapt XS from Waters. The column installed was a bioZen SEC-2 from Phenomenex with pure SiO_2 particles (1.8 µm particle size, 150 Å pore size, 150 x 2.1 mm) and an oven temperature of 20°C. 2 µL of sample was injected and the run was performed at an isocratic gradient of 35 µL/min with 50 mM ammonium acetate in water (LC-MS grade) for the native and 0.2 % formic acid in water (LC-MS grade) for the denatured measurements. After the chromatography the sample was ionized with an ESI source in positive mode. The source voltage was 3 kV, with a Cone Voltage of 50 V, a Source Offset of 20 V, at a Source Temperature of 70°C and Desolvation Temperature of 200°C. We set the Desolvation Gas Flow to 550 L/h, the Nebuliser gas flow to 6 bar and the cone gas flow to 100 L/h. The MS experiment was conducted without isolation or fragmentation in resolution mode of the TOF and a mass range of 100 to 5000 m/z. Before the measurements the instrument was calibrated with sodium iodide up to 5000 m/z. For the recalibration we used an internal recalibration with the analyte masses at different charge states.

Ion mobility mass spectrometry– native & denatured

The refolded proteins and recombinant protein stock solutions were buffer-exchanged four times in 200 mM ammonium acetate buffer (pH 7, LC-MS grade) at 14,000 x g for 30 min using 3k Amicon® Ultra centrifugal filters (Merck). The buffer-exchanged protein solutions were diluted with 200 mM ammonium acetate buffer to an initial concentration of 10 µM. The

samples were measured with direct-infusion nanoESI on a Synapt G2-S from Waters. 10 µL of protein solution was loaded in a self-pulled nanoESI capillary (Micropipette Puller Model P-97 from Sutter Instrument). The Capillary Voltage was between 1.0 and 2.0 kV, Sampling Cone 20 V, Source Offset 50 V, Source Temperature 30°C, Cone Gas Flow 200 L/h, and Purge Gas Flow 300 mL/h for the nanoESI source. For recalibration, we performed an internal calibration using the masses of the analytes with a first-order regression.

For the IM-MS measurements, we used helium at 180 mL/min as the cooling gas and nitrogen at 90 mL/min as the drift gas for the IMS cell. Wave velocities of 800, 1000 and 1150 m/s were used in the ion mobility cell. The wave height was 40 V each time with a constant wave velocity of 141 m/s and a wave height of 6 V in the transfer cell. The DC bias voltage of the trap was 30 V. The quadrupole with the RF generator of 800 kHz was set to RF only, and the TOF was in resolution mode. The mass range was from 400 to 5000 m/z. Before the measurements, the MS was mass calibrated with sodium iodide to 5000 m/z.

For CCS calibration, we measured bovine ubiquitin, equine cytochrome c, and equine holomyoglobin with the same settings. A logarithmic regression model was chosen for calibration (*10*). The peak value is the highest data point, while the weighted average is based on equation (1).

$$(1)\ Weighted\ Average\ CCS\ (\text{Å}^2) = \sum_{i=0}^{\infty} x_i * \frac{y_i}{\sum_{i=0}^{\infty} y_i}$$

Where x_i is the collision cross section associated with arrival time i and y_i is the corresponding intensity.

For the denatured IM-MS measurements the samples were diluted to a protein concentration of 100 nM with 0.2 % formic acid in 95:5 Water:Acetonitrile with (LC-MS grade).

The IM-MS measurements were performed with an HDX setup from Waters. This included a PAL RTC Autosampler from Leap, a UHPLC with µBinary Pump and Auxiliary Pump, the HDX Manager and a Synapt XS from Waters. The column installed was a Acquity UPLC Protein BEH C4 from Waters which is based on SiO_2 particles (1.7 µm particle size, 300 Å pore size, 50 x 2.1 mm) and an oven temperature of 25°C. 50 µL of sample was injected.

Eluent A was 0.2% formic acid in water (LC-MS grade) and eluent B was 0.2% formic acid in acetonitrile (LC-MS grade). The gradient started at 95% A and 5% B and was held for 1 min. Between 1 min and 5 min, it was increased to 50% B. Over the course of 5 to 10 min, 100% B was reached and held for 4 min. Within 6 s, the gradient was set back to 5% B for 6 min to re-equilibrate the column. Subsequently, the sample was ionized with an ESI source in positive

mode. The source voltage was 3 kV, with a Cone Voltage of 50 V, a Source Offset of 20 V, at a Source Temperature of 70°C and Desolvation Temperature of 200°C. We set the Desolvation Gas Flow to 550 L/h, the Nebuliser gas flow to 6 bar and the cone gas flow to 100 L/h. The Synapt XS uses traveling-wave ion mobility (*11, 12*). For the IM-MS measurements we used helium at around 3.4 mbar as cooling gas and nitrogen at 2.9 mbar as drift gas for the IMS-cell. Wave velocities of 500, 600 and 700 m/s were used. The wave height was held at 40 V with a constant wave velocity of 141 m/s and wave height of 6 V in the transfer cell. The trap DC bias was 45 V. The quadrupole with the RF generator of 300 kHz was set to RF only and the TOF was in resolution mode. The mass range was from 100 to 2000 m/z. Before the measurements the MS was mass calibrated with sodium iodide up to 5000 m/z. We used the LockSpray with leucine-enkephalin as a reference, where every minute for one second the reference was measured, with the masses 120.0813 m/z, 278.1141 m/z, 397.1876 m/z, 425.1825 m/z and 556.2771 m/z (*13*) with a first-order regression.

For the CCS calibration we measured with the same settings separate solutions with 10 µM protein in 200 mM ammonium acetate buffer of bovine ubiquitin, equine cytochrom c, and equine *holo*-myoglobin. The mobilograms were extracted with CIUSuite 2 (*14*). A logarithmic regression model was chosen for calibration (*10*). The top value is the highest data point, while the weighted average was based on Equation (1).

Protein Crystallization

Complexes were prepared by refolding F67E/K58 (i, i+9) or F67E/K60Orn (i, i+7) according to the protocol described earlier. After combining the fractions and exchanging the buffer to 20 mM HEPES pH 8, 20 mM NaCl and concentrating the samples using Amicon® Ultra 2 mL centrifugal filters, the samples were mixed with a slight excess of SAFit1 previously dissolved in 20 mM DMSO. The samples were purified by size exclusion chromatography in 20 mM HEPES pH 8, 20 mM NaCl and the fractions at an elution time of approx. 13.5 min were concentrated to a protein concentration of 3 mg/mL for F67E/K60Orn (i, i+7) and 1 mg/mL for F67E/K58 (i, i+9).

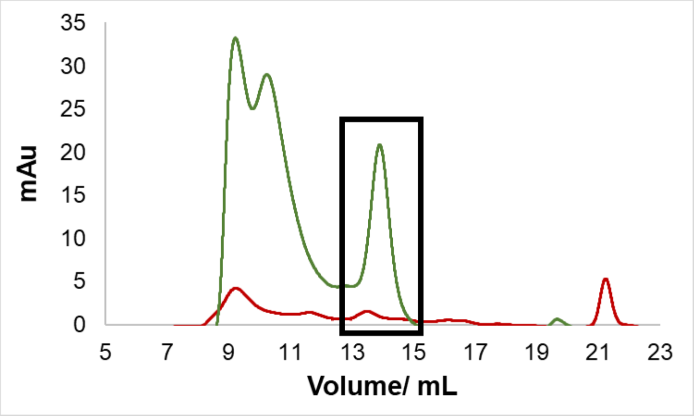

Figure SI1: Size exclusion chromatograms of cyclized variants in complex with SAFit1. Green chromatogram: F67E/K60Orn (i, i+7) and red chromatogram: F67E/K58 (i, i+9). Black box highlights the fraction which was used for protein crystallography after concentrating.

Crystallization was performed at room temperature using the hanging drop vapour-diffusion method, equilibrating mixtures of 1 µl protein complex and 1 µl reservoir against 500 µl reservoir solution. Crystals were obtained from reservoir solutions containing 32 % PEG-3350 for F67E/K58 (i, i+9) and 30 % PEG-3350 for F67E/K60Orn (i, i+7), 0.2 M NH_4-acetate, 0.1 M HEPES-NaOH pH 7.5. Crystals were fished, cryoprotected with 30% PEG-3350, 0.2 M NH_4-acetate, 0.1 M HEPES-NaOH pH 7.5 and 10% ethylene glycol and flash frozen in liquid nitrogen.

The crystallographic experiments were performed on the BL14.1 beamline at the Helmholtz-Zentrum BESSY II synchrotron, Berlin, Germany (*15*). Diffraction data were integrated with XDS and further processed with the implemented programs of the CCP4i and CCP4i2 interface (*16–19*). The data reduction was conducted with Aimless (*16, 20, 21*). Crystal structures were solved by molecular replacement using Phaser (*22*). Iterative model improvement and refinement were performed with Coot and Refmac5 (*23–28*). The dictionary for SAFit1 was generated with PRODRG implemented in CCP4i (*29*). The lactam bridge was generated with AceDRG (*30*). Residues facing solvent channels without detectable side chain density were truncated.

Table SI2: Refinement data.

PDB entry	8PJ8<br>FKBP51FK1 F67E/K60Orn<br>(i, i+7)<br>SAFit1	8PJA<br>FKBP51FK1 F67E/K58<br>(i, i+9)<br>SAFit1
**Data collection**		
Beamline	BESSY II (BL14.1)	BESSY II (BL14.1)
Wavelength	λ = 0.9184 Å	λ = 0.9184 Å
Space group	$P\ 2_1\ 2_1\ 2_1$	$P\ 2_1\ 2_1\ 2_1$
Cell dimensions		
a, b, c (Å)	45.22, 48.37, 57.20	42.06, 48.54, 56.03
α, β, γ (°)	90, 90, 90	90, 90, 90
Resolution (Å)	36.96-1.50 (1.53-1.50)	36.72-1.60 (1.63-1.60)
R_{merge}	0.058 (1.156)	0.083 (1.536)
R_{pim}	0.030 (0.621)	0.032 (0.801)
$I/\sigma(I)$	18.7 (1.9)	14.9 (1.4)
CC1/2	0.999 (0.729)	0.999 (0.527)
Completeness (%)	97.6 (97.1)	99.7 (99.0)
Redundancy	8.5 (8.2)	8.5 (8.7)
**Refinement**		
Resolution (Å)	36.96-1.50	36.72-1.60
No. of reflections	20089	15615
R_{work}/R_{free} (%)	19.3/21.6	19.6/21.9
Total number of atoms	2148	2068
Average B, all atoms (Å^2)	25.0	25.0
R.m.s. deviations		
Bond lengths (Å)	0.0122	0.0107
Bond angles (°)	1.794	1.751
Ramachandran plot		
Favoured	121 (98%)	119 (96%)
Allowed	2 (2%)	5 (4%)
Outlier	0	0

Protein production and purification

Chemically competent *E. coli* BL21 (DE3) cells were transformed with a plasmid containing the gene construct for FKBP51$^{16\text{-}140}$ (A19T, C103A, C107I, 16-140 AA) with an N-terminal His- and Sumo-Tag. Therefore, 10 ng of the plasmid was mixed with chemically competent *E.coli* BL21 (DE3) cells and incubated on ice for 30 min. The heat shock is performed for 45 s at 42°C, following the addition of 250 µL SOC media and an incubation of 2 h at 37°C. The preculture was prepared by adding the transformed cells to a flask containing 200 mL of LB media and 0.1 % v/v kanamycin which was incubated over night at 37°C and with 150 rpm. 4 L of LB media were inoculated to an OD_{600} of 0.1 with the preculture. As soon as a OD_{600} of approximately 0.5 was reached, the culture was induced by the addition of 1 mM isopropyl 1-thio-D-galactopyranoside and incubated over night at 25°C and with 150 rpm. The cells were harvested by centrifugation (10 000 g, 10min, 4°C) and stored at -20°C until lysis.

For the lysis, the cells were resuspended in 15 mL lysis buffer (20 mM HEPES pH 8, 300 mM NaCl) before 2 mM EDTA, 1 mM PMSF and 1 mg/mL Lysozym were added. The incubation time was 2 h at 4°C on a rolling device. Afterwards 0.1 mg/mL DNase and 100 mM $MgSO_4$ were added, and the tube is shaken at 4°C on a rolling device for another hour. Additionally, the samples are sonicated 3 x 3 min. Cellular debris was collected by centrifugation (13 000 g, 15 min, 4°C) and the supernatant was further purified with metal ion affinity chromatography (IMAC) to obtain the pure, His-tagged protein of interest (Equilibration Buffer: 20 mM HEPES pH 8, 500 mM NaCl; Washing Buffer: 20 mM HEPES pH 8, 500 mM NaCl, 10 mM imidazole; Elution Buffer: 20 mM HEPES pH 8, 500 mM NaCl, 300 mM imidazole). To remove the imidazole, the fractions with the highest protein concentration (measured photometrically at A_{280}) were combined and desalted using the HiPrep 26/10 Desalting column on an Äkta Pure Protein Purification System (Cytiva) at a flow rate of 5 mL/min with the Equilibration Buffer (20 mM HEPES pH 8, 500 mM NaCl).The His- and Sumo-Tag were removed by the addition of Sumo protease (1:100 (m/m)) and incubation over night at 4°C on a rolling device. To filter the cleaved His- and Sumo-Tag out of the sample, a reverse IMAC was performed, collecting the supernatant. To achieve a higher purity, the sample was also purified by SEC using the HiLoad 16/600 Superdex 75 pg column with a flow rate of 0.5 mL/min (Buffer: 20 mM HEPES pH 8, 20 mM NaCl). The fractions containing the pure protein were concentrated using Amicon® Ultra 2 mL centrifugal filter. The protein was aliquoted and stored at -80°C.

Fluorescence polarization

To determine the binding affinity of our low affinity tracer (Fluorescein conjugated SAFit1-analogue, without Top-group) and the respective protein variants, fluorescence polarization binding assays were performed. In a 384-well assay plate (black, flat bottom, low-binding) a serial dilution of the protein variants under investigation were prepared in FP-Assay Buffer (20 mM HEPES pH 8, 150 mM NaCl, 0.015 % Triton X-100). In case of an active site titration the tracer was added at an end concentration of 30 nM, and 1 nM for recording binding curves. The plates were incubated for 30 min at room temperature and the fluorescence polarization was measured on a plate reader. The fluorescence polarization values were normalized with respect to the highest signal.

The 4-parameter fitting equation was used to determine the EC_{50} from the active site titration, defined here as the total concentration at which free and bound tracer are present in equal quantities:

$$y = \min + \frac{(max - min)}{1 + \left(\frac{x}{EC_{50}}\right)^{-Hillslope}}$$

min= lower plateau
max= upper plateau
EC_{50}= half maximal effective concentration

For the binding curves, the protein solutions were used undiluted with a protein concentration adjusted by the previously performed active site titration according to the following equation:

$$c_{AST,protein} = \frac{c_{Tracer} \cdot c_{UV,protein}}{2 \cdot EC_{50}}$$

$c_{AST,protein}$= concentration of the protein determined by active site titration
$c_{UV,protein}$ = concentration of the protein determined by UV
c_{Tracer} = concentration of tracer
EC_{50}= half maximal effective concentration

To determine the binding affinity of our tracer with the respective protein variants, the recorded binding curves were fitted by a model which was described by Wang et. al in 1992 (*31*).

$$tracer\ bound = \frac{100}{Lt} \cdot 0.5 \cdot (Pt + Lt + KD - \sqrt{(Pt + Lt + KD)^2 - 4 \cdot LtPt})$$

L_t= total concentration of the tracer
R_t= total concentration of the Protein
K_D= binding constant of complex PL

Structures of the tracers:

A

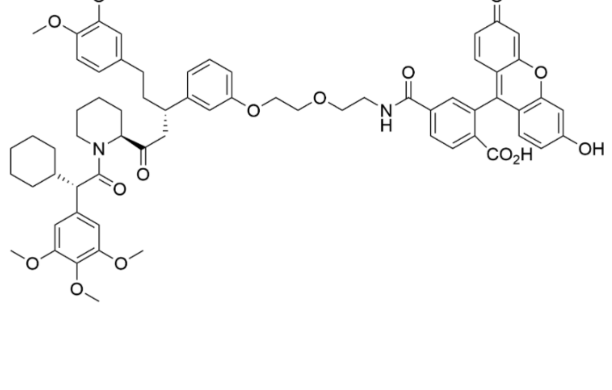

B

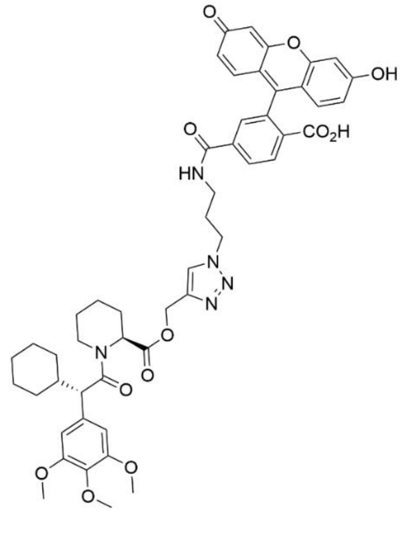

Figure SI2: Structures of the used tracers. A: SAFit1 based tracer coupled to fluorescein. B: Low affinity tracer (without top group) coupled to fluorescein.

Synthesis of low affinity tracer (4-((3-(4-((((S)-1-((S)-2-Cyclohexyl-2-(3,4,5-trimethoxyphenyl)acetyl)piperidine-2-carbonyl)oxy)methyl)-1H-1,2,3-triazol-1-yl)propyl)carbamoyl)-2-(6-hydroxy-3-oxo-3H-xanthen-9-yl)benzoic acid)

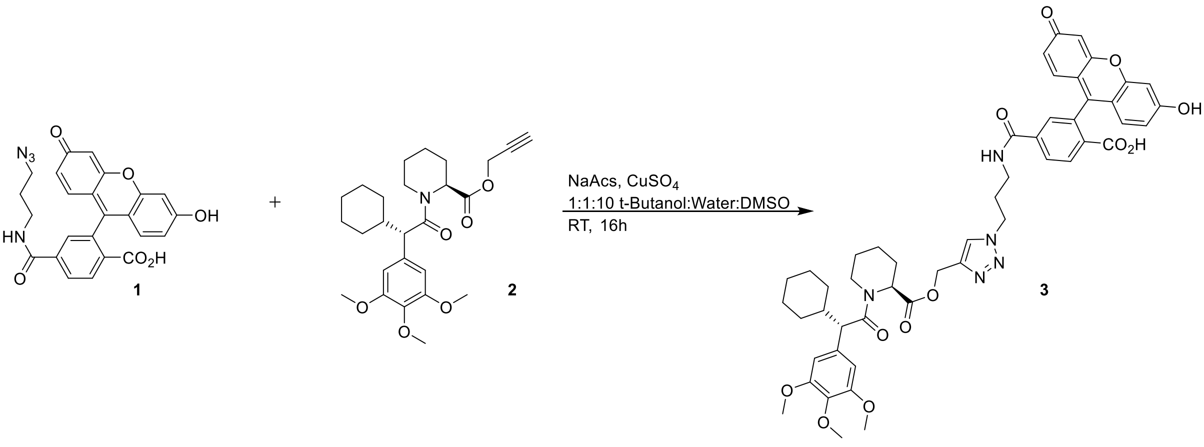

Scheme SI 1:Synthesis of low-affinity tracer.

4-((3-Azidopropyl)carbamoyl)-2-(6-hydroxy-3-oxo-3H-xanthen-9-yl)benzoic acid (6-carboxy fluorescein azide, Roth, **1**) (10.01 mg, 22 µmol, 1.00 eq.), prop-2-yn-1-yl (S)-1-((S)-2-cyclohexyl-2-(3,4,5-trimethoxyphenyl)acetyl)piperidine-2-carboxylate (**2**) (9.99 mg, 22 µmol, 1 eq.), sodium ascorbate (4.33 mg, 22 µmol, 1 eq.) and copper sulfate (5.45 mg, 22 µmol, 1 eq.) were dissolved in 1:1:1 tert-butanol:water:DMSO and stirred for 16 h at room temperature under argon atmosphere. The reaction solution was afterwards extracted with ethyl acetate (3 x 10 mL) and washed once with brine (50 mL) before combining and drying the organic layers with $MgSO_4$. After solvent removal under reduced pressure, the crude product was purified by RP-HPLC on an Interchim puriFlash 5.250 system fitted with a preperative LC column (Luna-5-C182-250X2, C18). The run was performed with a flow rate of 16 mL/min and a 40-70 %B within 6 CV (column volume) gradient, where solvent A was 0.1 % TFA in water and solvent B was 0.1 % TFA in acetonitrile. The fractions with the pure product were combined and the solvent was removed under reduced pressure and lyophilized. A yellow solid was obtained (**3**).

Yield: 14.8 mg (16.17 µmol, 74 %)

LC-MS (5 – 100 % solvent B, 2 min): R_t = 2.089 min, purity (220 nm): 99 %, purity (254 nm): 87 % m/z: calculated = 916.38 [M+H]+, found = 916.40 [M+H]+.

HRMS (ESI): calculated = 916.37635[M+H]+, found = 916.37690 [M+H]+, error [ppm] = 0.6.

Additional Data

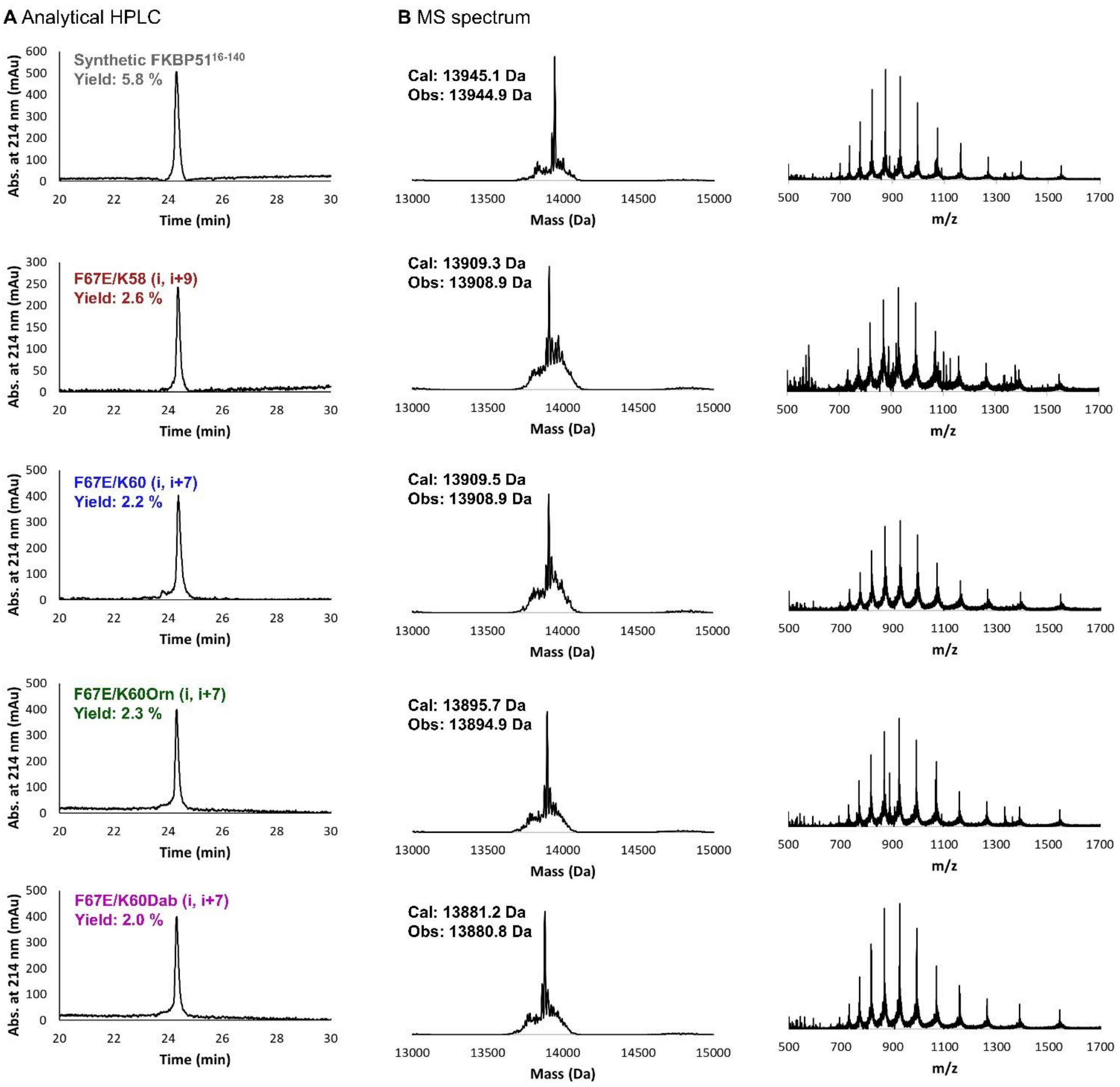

Figure SI3: Zoom in A: Analytical HPLC and B: MS spectrum (left: deconvoluted mass spectrum, right: m/z spectrum) of synthesized protein variants.

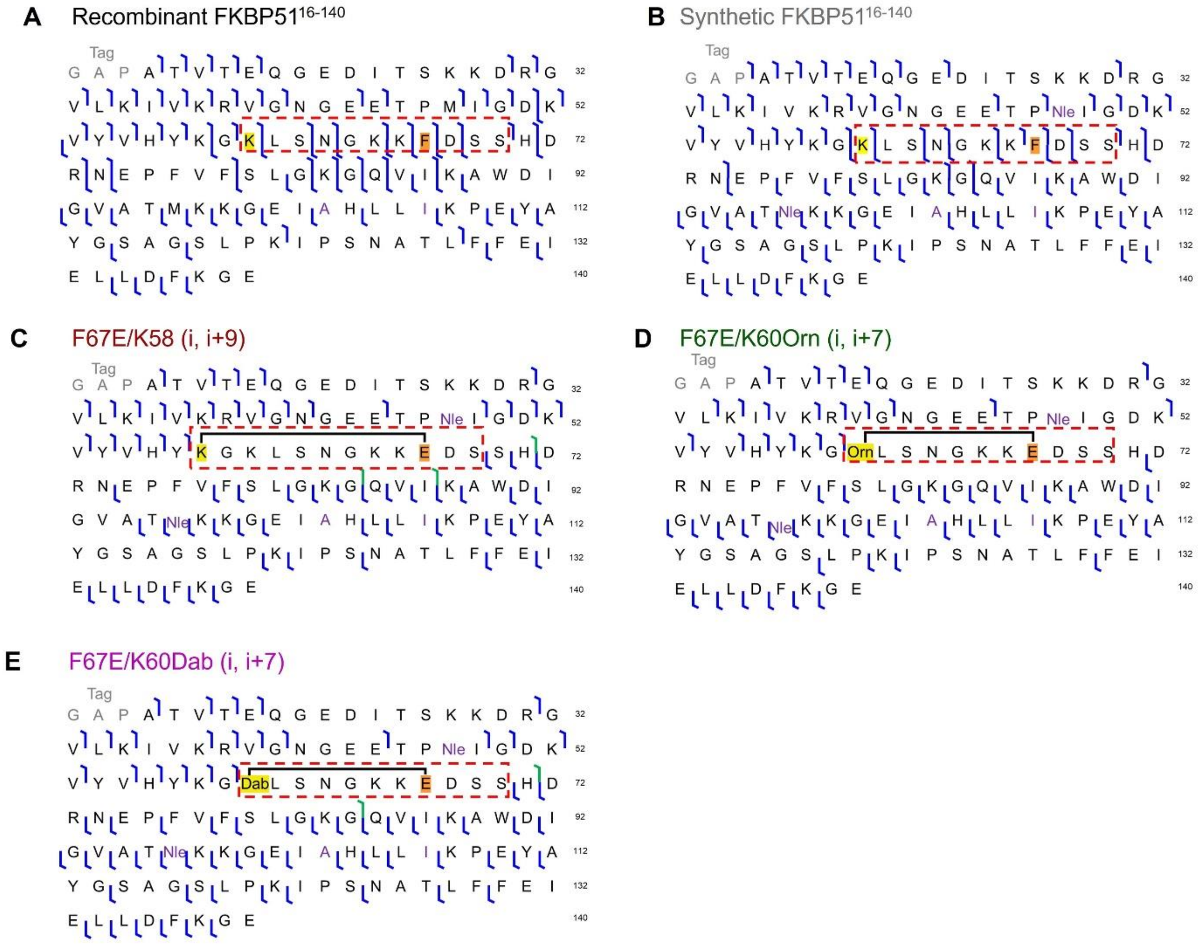

Figure SI4: ETD Fragmentation map from top-down measurements. ETD fragmentation map from top-down measurements of recombinant FKBP51$^{16\text{-}140}$ (A), synthetic FKBP51$^{16\text{-}140}$ (B), F67E/K58 (i, i+9) (C), F67E/K60Orn (i, i+7) (D) and F67E/K60Dab (i, i+7) (E). The numbers on the right side correspond to the FKBP51$^{16\text{-}140}$ sequence. A-E: Typical c- or z-fragments for ETD are indicated in blue. In green are the c-fragments with an H_2O-loss. The FKBP51 sequence starts with the alanine residue in position 16. The purple amino acids indicate the modifications C103A and C107I as well as the exchange of methionine in norleucine (Nle) in positions 48 and 97. Positions 60 and 58 are highlighted in yellow, position 67 in orange, and the black line indicates the formed lactam-bridge.

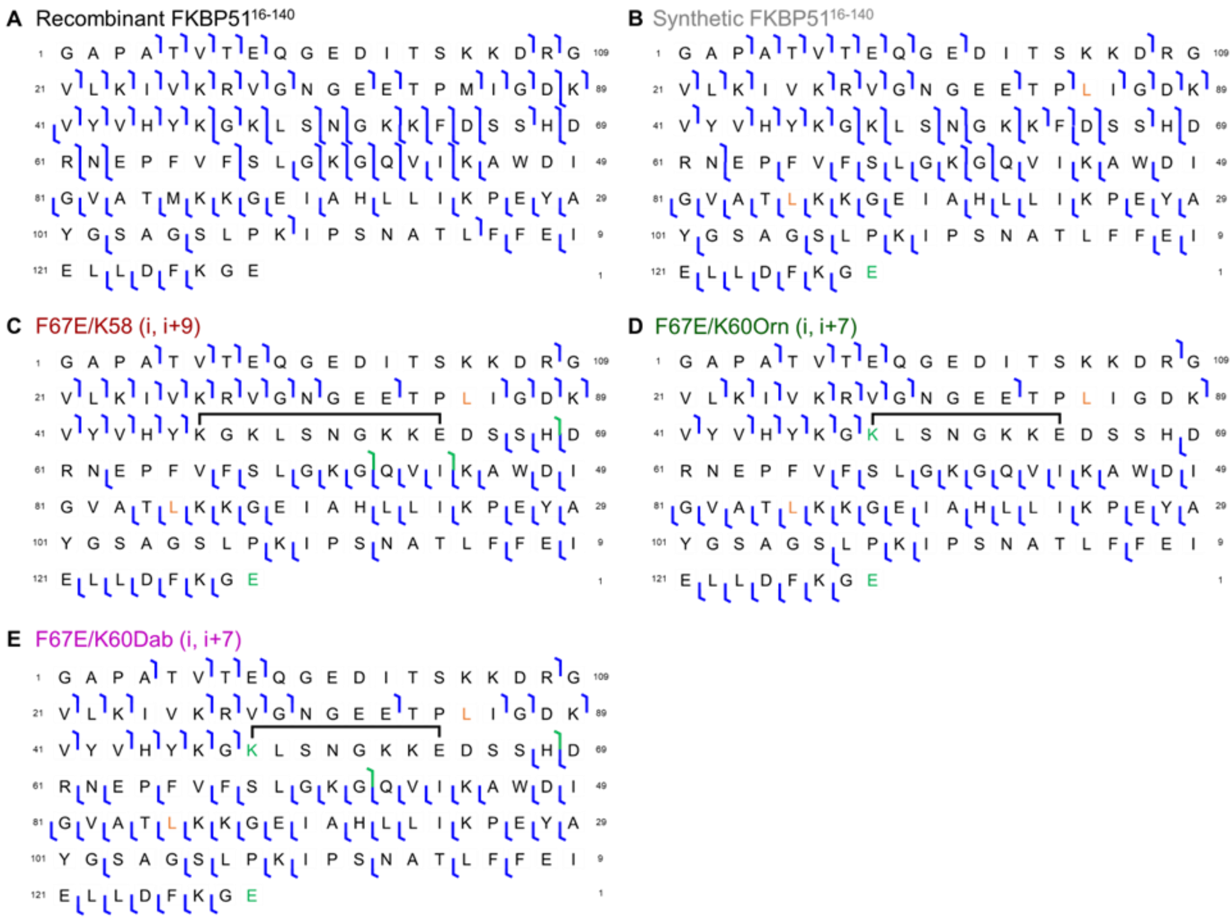

Figure SI5: ETD Fragmentation map from top-down measurements. Note that the data in this Figure are the same as in Figure SI4, but the residue numbering follows that of the FKBP51$^{16\text{-}140}$ construct, rather than that of the full-length protein. Numbers on the left count up, while numbers on the right count down, in order to facilitate tracking the cleavage position associated with c- and z-fragments, respectively. A: ETD fragment map of recombinant FKBP51$^{16\text{-}140}$, with a sequence coverage of 100 %, a residue cleavage of 62.50 %, and a total number of 49 c-fragments and 44 z-fragments. B: ETD fragment map of synthetic FKBP51$^{16\text{-}140}$, with a sequence coverage of 100 %, a residue cleavage of 66.41 %, and a total number of 40 c-fragments and 50 z-fragments. L36 and L85 are norleucine. C: ETD fragment map of F67E/K58 (i, i+9), with sequence coverage of 90.6%, residue cleavage of 50.00 %, and a total of 25 c-fragments and 39 z-fragments in blue. L36 and L85 are norleucine. Shown in green are the c-fragments with a loss of H_2O (- 18.0153 Da). Including these increases the c-fragments to 28 and the sequence coverage to 100 %. The black line indicates the lactam bridge formed. D: ETD fragment map of F67E/K60Orn (i, i+7), with sequence coverage of 90.60 %, residual cleavage of 47.65 %, and total number of 20 c-fragments and 41 z-fragments in blue. L36 and L85 are norleucine. K48 has a mass difference of - 14.0156 Da as a result of the replacement of lysine by ornithine. The black line indicates the lactam bridge formed. E: ETD fragment map of F67E/K60Dab (i, i+7), with sequence coverage of 91.40%, residue cleavage of 55.47 %, and total number of 22 c-fragments and 49 z-fragments in blue. L36 and L85 are norleucine. K48 has a mass difference of - 28.0313 Da because of the replacement of lysine by diaminobutyric acid. Shown in green are the c-fragments with a loss of H_2O (- 18.0153 Da). Including these increases the number of c-fragments to 24 and the sequence coverage to 100 %. The black line indicates the lactam bridge formed. For B-E a mass difference of -0.984 Da is observed since the C-terminus is an amide due to synthesis instead of a carboxylic acid.

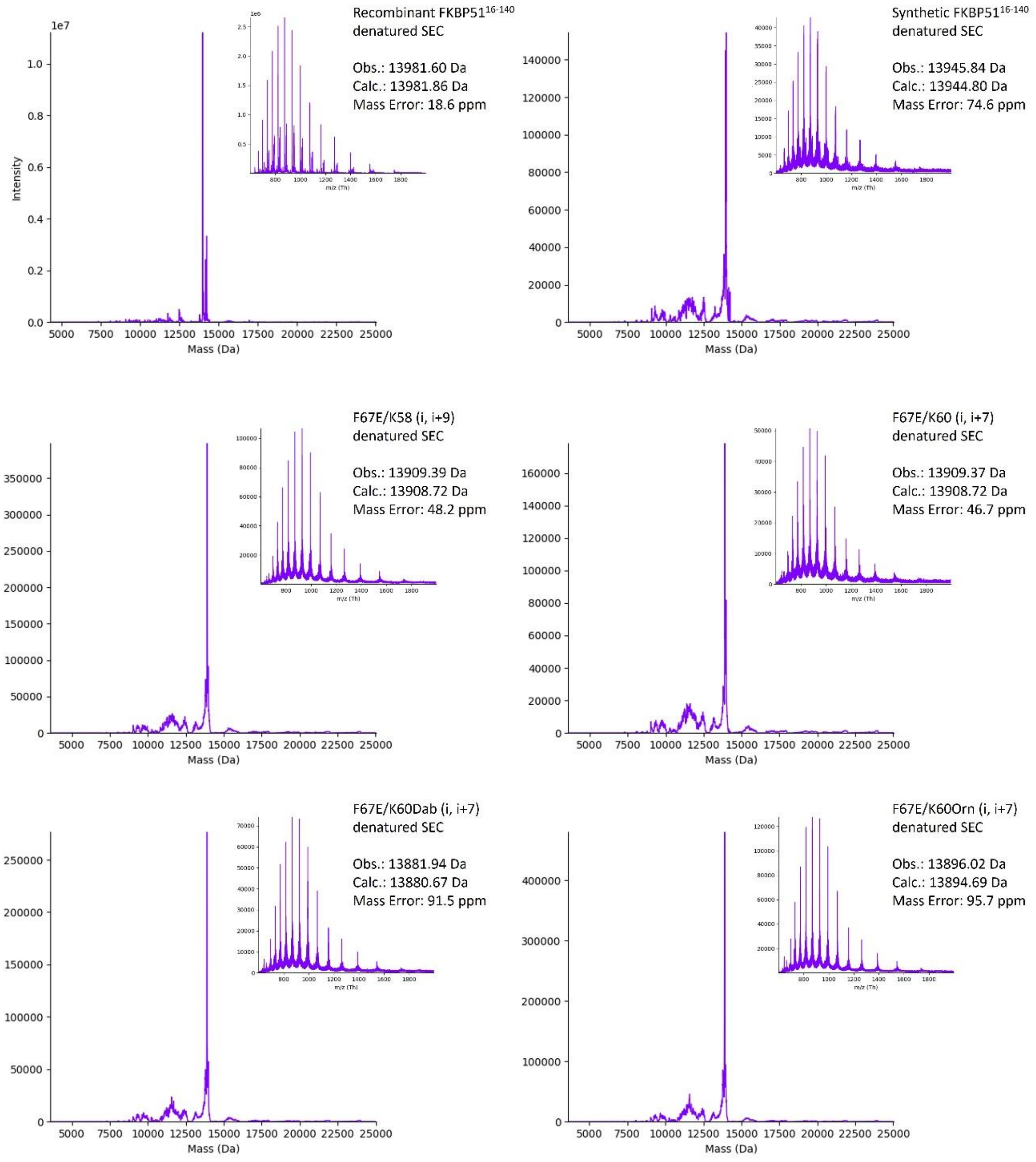

Figure SI6: MS spectrum of all variants analyzed in the SEC-MS experiment (size exclusion chromatography mass spectrometry) under denaturing conditions.

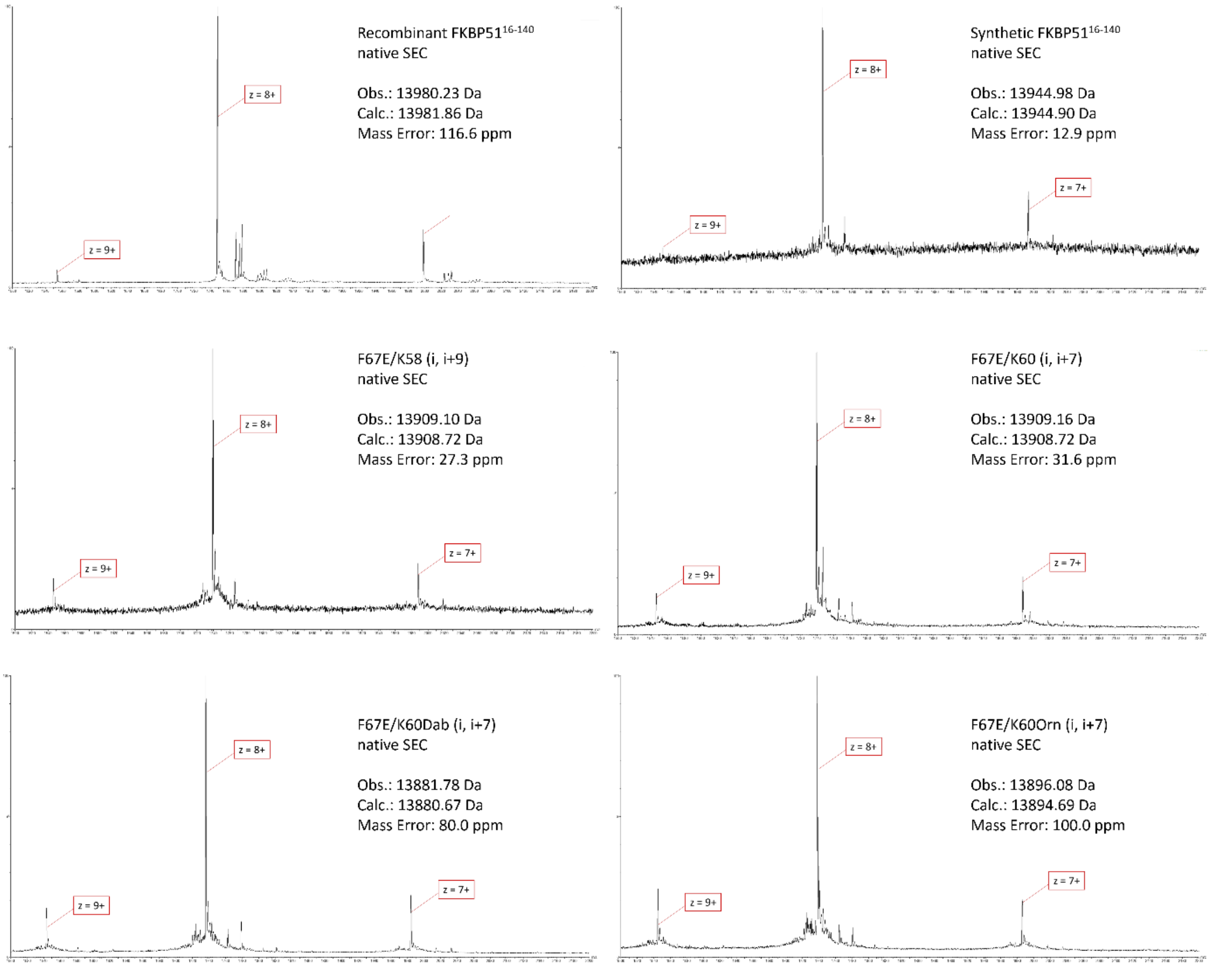

Figure SI7: MS spectrum of all variants analyzed in the SEC-MS experiment (size exclusion chromatography mass spectrometry) under native conditions.

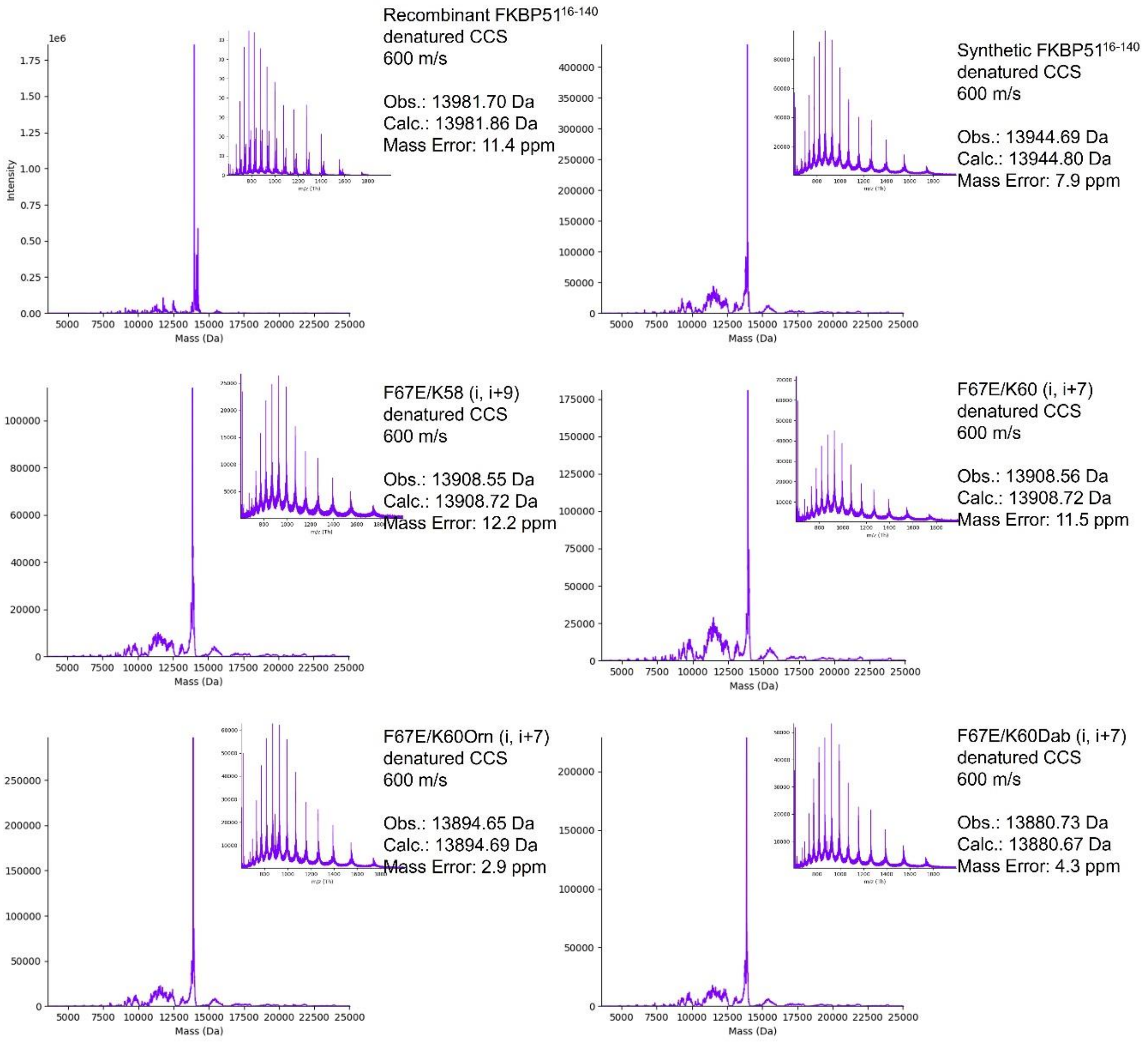

Figure SI8: Intact MS spectra from denatured CCS measurements for all variants.

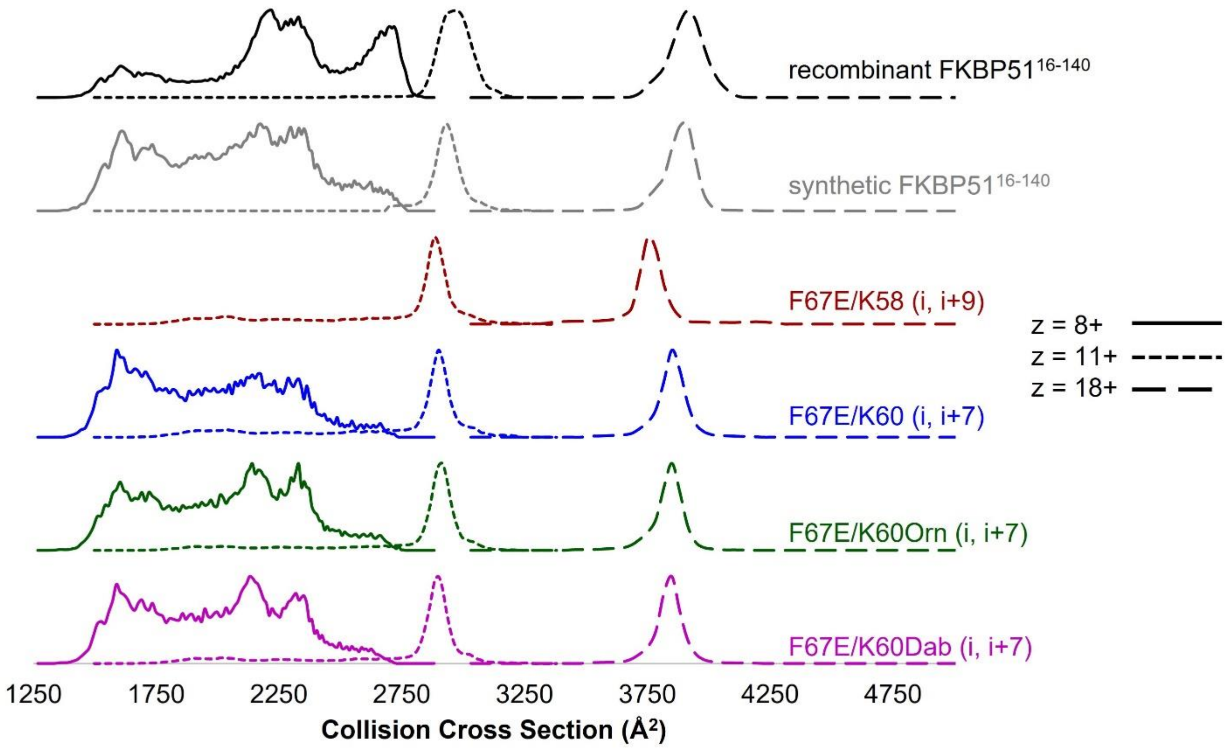

Figure SI 9: Mobilograms of the recombinant, synthetic FKBP51$^{16\text{-}140}$ and lactam-bridged variants at charge state 8+, 11+ and 18+ under denaturing conditions. The collision cross section increases with a higher charge state, showing a similar denaturing behavior for all variants.

References

1. Mijalis, A. J.; Thomas III, D. A.; Simon, M. D.; Adamo, A.; Beaumont, R.; Jensen, K. F.; Pentelute, B. L. A fully automated flow-based approach for accelerated peptide synthesis. *Nature chemical biology* **2017,** *13* (5), 464–466.

2. Hartrampf, N.; Saebi, A.; Poskus, M.; Gates, Z. P.; Callahan, A. J.; Cowfer, A. E.; Hanna, S.; Antilla, S.; Schissel, C. K.; Quartararo, A. J. Synthesis of proteins by automated flow chemistry. *Science* **2020,** *368* (6494), 980–987.

3. Bracher, A.; Kozany, C.; Thost, A.-K.; Hausch, F. Structural characterization of the PPIase domain of FKBP51, a cochaperone of human Hsp90. *Acta crystallographica. Section D, Biological crystallography* **2011,** *67* (6), 549–559.

4. http://db.systemsbiology.net/proteomicsToolkit/FragIonServlet.html. *Proteomics Toolkit*: *Institute for Systems Biology, Seattle, WA, USA;*

5. Wu, Z.; Roberts, D. S.; Melby, J. A.; Wenger, K.; Wetzel, M.; Gu, Y.; Ramanathan, S. G.; Bayne, E. F.; Liu, X.; Sun, R. MASH explorer: a universal software environment for top-down proteomics. *Journal of proteome research* **2020,** *19* (9), 3867–3876.

6. Reid, D. J.; Diesing, J. M.; Miller, M. A.; Perry, S. M.; Wales, J. A.; Montfort, W. R.; Marty, M. T. MetaUniDec: high-throughput deconvolution of native mass spectra. *Journal of the American Society for Mass Spectrometry* **2018,** *30* (1), 118–127.

7. Horn, D. M.; Zubarev, R. A.; McLafferty, F. W. Automated reduction and interpretation of. *Journal of the American Society for Mass Spectrometry* **2000,** *11* (4), 320–332.

8. Syka, J. E. P.; Coon, J. J.; Schroeder, M. J.; Shabanowitz, J.; Hunt, D. F. Peptide and protein sequence analysis by electron transfer dissociation mass spectrometry. *Proceedings of the National Academy of Sciences* **2004,** *101* (26), 9528–9533.

9. ROEPSTORFE, P. Proposal for a common nomenclature for sequence ions in mass spectra of peptides. *Biomed. Mass Spectrom.* **1984,** *11*, 601–605.

10. Ruotolo, B. T.; Benesch, J. L. P.; Sandercock, A. M.; Hyung, S.-J.; Robinson, C. V. Ion mobility–mass spectrometry analysis of large protein complexes. *Nature protocols* **2008,** *3* (7), 1139–1152.

11. Shvartsburg, A. A.; Smith, R. D. Fundamentals of traveling wave ion mobility spectrometry. *Analytical chemistry* **2008,** *80* (24), 9689–9699.

12. Pringle, S. D.; Giles, K.; Wildgoose, J. L.; Williams, J. P.; Slade, S. E.; Thalassinos, K.; Bateman, R. H.; Bowers, M. T.; Scrivens, J. H. An investigation of the mobility separation of some peptide and protein ions using a new hybrid quadrupole/travelling wave IMS/oa-ToF instrument. *International Journal of Mass Spectrometry* **2007,** *261* (1), 1–12.

13. Sztáray, J.; Memboeuf, A.; Drahos, L.; Vékey, K. Leucine enkephalin—a mass spectrometry standard. *Mass spectrometry reviews* **2011,** *30* (2), 298–320.

14. Polasky, D. A.; Dixit, S. M.; Fantin, S. M.; Ruotolo, B. T. CIUSuite 2: Next-generation software for the analysis of gas-phase protein unfolding data. *Analytical chemistry* **2019,** *91* (4), 3147–3155.

15. Gerlach, M.; Mueller, U.; Weiss, M. S. The MX beamlines BL14. 1-3 at BESSY II. *Journal of large-scale research facilities JLSRF* **2016,** *2*, A47-A47.

16. Potterton, L.; Agirre, J.; Ballard, C.; Cowtan, K.; Dodson, E.; Evans, P. R.; Jenkins, H. T.; Keegan, R.; Krissinel, E.; Stevenson, K. CCP4i2: the new graphical user interface to the CCP4 program suite. *Acta Crystallographica Section D: Structural Biology* **2018,** *74* (2), 68–84.

17. Potterton, E.; Briggs, P.; Turkenburg, M.; Dodson, E. A graphical user interface to the CCP4 program suite. *Acta crystallographica. Section D, Biological crystallography* **2003,** *59* (7), 1131–1137.

18. Project, C. C. The CCP4 suite: programs for protein crystallography. *Acta crystallographica. Section D, Biological crystallography* **1994,** *50* (Pt 5), 760–763.

19. Winn, M. D.; Ballard, C. C.; Cowtan, K. D.; Dodson, E. J.; Emsley, P.; Evans, P. R.; Keegan, R. M.; Krissinel, E. B.; Leslie, A. G. W.; McCoy, A. Overview of the CCP4 suite and current developments. *Acta crystallographica. Section D, Biological crystallography* **2011,** *67* (4), 235–242.

20. Evans, P. R.; Murshudov, G. N. How good are my data and what is the resolution? *Acta crystallographica. Section D, Biological crystallography* **2013,** *69* (7), 1204–1214.

21. Evans, P. R. An introduction to data reduction: space-group determination, scaling and intensity statistics. *Acta crystallographica. Section D, Biological crystallography* **2011,** *67* (4), 282–292.

22. McCoy, A. J.; Grosse-Kunstleve, R. W.; Adams, P. D.; Winn, M. D.; Storoni, L. C.; Read, R. J. Phaser crystallographic software. *Journal of applied crystallography* **2007,** *40* (4), 658–674.

23. Vagin, A. A.; Steiner, R. A.; Lebedev, A. A.; Potterton, L.; McNicholas, S.; Long, F.; Murshudov, G. N. REFMAC5 dictionary: organization of prior chemical knowledge and guidelines for its use. *Acta crystallographica. Section D, Biological crystallography* **2004,** *60* (12), 2184–2195.

24. Emsley, P.; Lohkamp, B.; Scott, W. G.; Cowtan, K. Features and development of Coot. *Acta crystallographica. Section D, Biological crystallography* **2010,** *66* (4), 486–501.

25. Murshudov, G. N.; Skubák, P.; Lebedev, A. A.; Pannu, N. S.; Steiner, R. A.; Nicholls, R. A.; Winn, M. D.; Long, F.; Vagin, A. A. REFMAC5 for the refinement of macromolecular crystal structures. *Acta crystallographica. Section D, Biological crystallography* **2011,** *67* (4), 355–367.

26. Murshudov, G. N.; Vagin, A. A.; Dodson, E. J. Refinement of macromolecular structures by the maximum-likelihood method. *Acta Crystallographica Section D: Biological Crystallography* **1997,** *53* (3), 240–255.

27. Nicholls, R. A.; Long, F.; Murshudov, G. N. Low-resolution refinement tools in REFMAC5. *Acta crystallographica. Section D, Biological crystallography* **2012,** *68* (4), 404–417.

28. Winn, M. D.; Murshudov, G. N.; Papiz, M. Z. Macromolecular TLS refinement in REFMAC at moderate resolutions; Elsevier, 2003, pp 300–321.

29. van Aalten, D. M. F.; Bywater, R.; Findlay, J. B. C.; Hendlich, M.; Hooft, R. W. W.; Vriend, G. PRODRG, a program for generating molecular topologies and unique

molecular descriptors from coordinates of small molecules. *Journal of computer-aided molecular design* **1996,** *10*, 255–262.

30. Long, F.; Nicholls, R. A.; Emsley, P.; Gražulis, S.; Merkys, A.; Vaitkus, A.; Murshudov, G. N. AceDRG: a stereochemical description generator for ligands. *Acta Crystallographica Section D: Structural Biology* **2017,** *73* (2), 112–122.
31. Wang, Z.-X.; Kumar, N. R.; Srivastava, D. K. A novel spectroscopic titration method for determining the dissociation constant and stoichiometry of protein-ligand complex. *Analytical biochemistry* **1992,** *206* (2), 376–381.
