# Supplementary material for: Automated Flow Peptide Synthesis Enables Engineering of Proteins with Stabilized Transient Binding Pockets
Source: ACS Cent Sci. 2024 Feb 28;10(3):649–57. doi: 10.1021/acscentsci.3c01283 (PMC10979424; doi:10.1021/acscentsci.3c01283)
Supplement: Supplementary file 2 — oc3c01283_si_002.pdf [file oc3c01283_si_002.pdf]

oc-2023-01283m.R1

Name: Peer Review Information for "Automated flow peptide synthesis enables engineering of proteins with stabilized transient binding pockets"

First Round of Reviewer Comments

Reviewer: 1

Comments to the Author

This study by Hausch and co-workers describes the chemical synthesis of FKBP51 that is locked into a rarely populated conformation. This conformation is relevant and of interest for the development of selective FKBP51 inhibitors (selective over FKBP52). For locking the conformation, they "tied" Phe67 of the conformatoinally mobile region to a surface residue. For linking Phe67 with specific amino acid side chains of FKBP, they produced the entire protein of 128 amino acids by chemical synthesis, which allowed flexible cross-linking of the two residues, Phe67 that was replaced with Glu, and Lys (or Orn, or Dab) via a lactam bridge. The chemical synthesis of such a large polypeptide (128 aa here) and cyclization (lactam) is usually challenging but the authors managed to do this efficiently by taking advantage of recently established methods for flow synthesis of entire proteins, and they formed the lactam bridge on the solid phase by choosing suitable protecting groups. The authors demonstrated the correct folding by functional assays (binding site titration) and by X-ray crystallography, and they showed that locking into the rare conformation enhances substantially the affinity for ligands that bind to the cryptic site.

The work is of interest as the strategy is elegant and much easier than established alternatives (e.g. genetic encoding, NCL). The data and finding appear robust, in particular giving the X-ray structures with clear electron density for the installed linkers. The methods of flow synthesis are not new, but exploiting it for locking proteins in a specific conformation is. I could imagine that this example and the strategy will be copied by many groups to apply flow chemistry for synthesizing entire proteins in which specific sites are cross-liked, for stabilizing protein conformations or similar applications. I can recommend publication, but also encourage to address the following questions that came up when I read the work.

Points to address:

1. MS analysis: the authors analyzed if the lactam bridge was correctly installed by MS (ETD fragmentation). The result of this experiment is Fig 2c, which was difficult for me to assess for its quality and robustness. It is not so clear when a fragment is considered to be "identified". In this method, there is always background and there will certainly be false positives and false negatives. I recommend to describe the results more quantitatively in the main text. Or potentially use a more quantitative method

(e.g. trypsination and MS analysis). Overall, I have no doubts that the linker is correctly installed due to the clear X-ray structure data.

2. Size exclusion chromatography: I recommend to show some protein size standards as reference, to allow analyzing if the recombinantly expression protein and the syntehsized and folded one have the expected size. I guess the authors have run them already but do not show them. Or if not, I recommend to repeat this.

3. Overall strategy: when seeing the strategy, I was immediately thinking that this could also be done by mutation of the same amino acid positions to Cys, recombinant expression, and cross-linking them by selective reaction (e.g. bis-electrophiles). I recommend to discuss this option. Are there cysteines in this protein that would prevent this? Or has it even been done and did not work?

Reviewer: 2

Comments to the Author

The submitted manuscript "Automated flow peptide synthesis enables engineering of

proteins with stabilized transient binding pockets" by Anna Charalampidou, Thomas Nehls, Christian Meyners, Satish Gandhesiri, Sebastian Pomplun, Bradley L. Pentelute, Frederik Lermyte and Felix Hausch demonstrates the use of chemical protein synthesis to produce conformationally locked derivatives of FKBP51. Furthermore, they show an improved affinity of conformationally specific ligands to these locked, synthetic derivatives. The manuscript is well written and provides an excellent example of the use of synthetic proteins in medicinal chemistry studies. However, there are some concerns regarding the analytical data that need to be addressed prior to publication of this study.

Major edits:

- The analytical data of the synthetic proteins (analytical HPLC and HR-LCMS) should be provided in both, the figure and the SI. In the SI it is currently missing, and in the manuscript figure the data is barely readable. Please enlarge analytical data in the manuscript and provide a full analytical data set in the SI.

- The authors claim that all samples have high purity, however, the 3rd derivative (and to some degree also the 5th) in Figure 2 show some larger impurities. Please comment on these.

Minor edits:

- In the abstract, the sentence "prepare proteins in a specific conformation" requires modification. The proteins are prepared with non-canonical amino acids enforcing this specific conformation, but the conformation is not prepared itself.

- In the abstract: I think "solid phase late-stage modifications" should be " solid-phase late-stage modifications" (as in solid-phase peptide synthesis)

- in previous reports, AFPS has been used as an abbreviation for "automated fast-flow peptide synthesis" and here it is "automated flow peptide synthesis". For consistency, I would recommend to insert the "fast" to keep the original meaning.

- For ion mobility MS (Figure 3C): please also show the "unfolded" control

- Supporting information: resin loading is missing in the materials section

Author's Response to Peer Review Comments:

Dear Dr. Editor,

we thank you and the reviewers for the constructive comments on our manuscript on the generation of conformationally stabilized FKBP51 variants by automated flow peptide synthesis. Please find attached the revised manuscript and SI in the clean version and in tract mode as well as a summary of the revision, in which we address the points raised by the reviewers point-by-point.

As the main revisions, we have improved the presentation of the analytical data of the generated constructs and added additional MS measurements in the supporting information. We previously also had explored crosslinking by bis-electrophiles, as suggested by reviewer 1, which we now mentioned in the manuscript. However, since this strategy turned out to be clearly inferior, we did not elaborated it further in the current manuscript.

Thank you very much in advance for re-considering our manuscript.

Best regards,

Felix Hausch

Revision summary for manuscript oc-2023-01283m

We thank the reviewers for their positive feedback on our work, the recognition of its importance, and the valuable suggestions for further improving our study.

We addressed the reviewer comments as follows:

------------------------------------ Reviewer(s)'
Comments to Author:
Reviewer: 1

Recommendation: Publish in ACS Central Science after minor revisions noted.

Comments:
This study by Hausch and co-workers describes the chemical synthesis of FKBP51 that is locked into a rarely populated conformation. This conformation is relevant and of interest for the development of selective FKBP51 inhibitors (selective over FKBP52). For locking the conformation, they "tied" Phe67 of the conformatoinally mobile region to a surface residue. For linking Phe67 with specific amino acid side chains of FKBP, they produced the entire protein of 128 amino acids by chemical synthesis, which allowed flexible cross-linking of the two residues, Phe67 that was replaced with Glu, and Lys (or Orn, or Dab) via a lactam bridge. The chemical synthesis of such a large polypeptide (128 aa here) and cyclization (lactam) is usually challenging but the authors managed to do this efficiently by taking advantage of recently established methods for flow synthesis of entire proteins, and they formed the lactam bridge on the solid phase by choosing suitable protecting groups. The authors demonstrated the correct folding by functional assays (binding site titration) and by X-ray crystallography, and they showed that locking into the rare conformation enhances substantially the affinity for ligands that bind to the cryptic site.

The work is of interest as the strategy is elegant and much easier than established alternatives (e.g. genetic encoding, NCL). The data and finding appear robust, in particular giving the X-ray structures with clear electron density for the installed linkers. The methods of flow synthesis are not new, but exploiting it for locking proteins in a specific conformation is. I could imagine that this example and the strategy will be copied by many groups to apply flow chemistry for synthesizing entire proteins in which specific sites are cross-liked, for stabilizing protein conformations or similar applications. I can recommend publication, but also encourage to address the following questions that came up when I read the work.

Points to address:
1. MS analysis: the authors analyzed if the lactam bridge was correctly installed by MS (ETD fragmentation). The result of this experiment is Fig 2c, which was difficult for me to assess for its

quality and robustness. It is not so clear when a fragment is considered to be "identified". In this method, there is always background and there will certainly be false positives and false negatives. I recommend to describe the results more quantitatively in the main text. Or potentially use a more quantitative method (e.g. trypsination and MS analysis). Overall, I have no doubts that the linker is correctly installed due to the clear X-ray structure data.

Thank you for this comment. Indeed, in top-down ETD, signal-to-noise ratios are often rather low, as the signal intensity is divided among a large number of fragments. However, there are established procedures for limiting the number of false-positive identifications, (i) use of instruments with high mass accuracy (such as the Synapt XS used in this work) and, (ii) the observation of several sequential isotope peaks (see *e.g.*, F. Lermyte et al., *J Am Soc Mass Spectrom*, 2014, 25, 343-350). We summarize our assignment procedure in the Supporting Information under "ETD", and have added the sentence: "In addition, we made sure that at least 4 isotope peaks of a fragment were present for it to be identified."

2. Size exclusion chromatography: I recommend to show some protein size standards as reference, to allow analyzing if the recombinantly expression protein and the syntehsized and folded one have the expected size. I guess the authors have run them already but do not show them. Or if not, I recommend to repeat this.

We did not use a protein size standard because in this case SEC is directly coupled to MS, which confirmed the correct mass of the synthesized and recombinantly expressed proteins. Please find now the respective data in the SI figures 6&7.
The main purpose of this experiment was to investigate the correct folding of the proteins. For this reason, the denatured proteins were included as references.

3. Overall strategy: when seeing the strategy, I was immediately thinking that this could also be done by mutation of the same amino acid positions to Cys, recombinant expression, and cross-linking them by selective reaction (e.g. bis-electrophiles). I recommend to discuss this option. Are there cysteines in this protein that would prevent this? Or has it even been done and did not work?

This is an excellent idea. Indeed, we have tried to use homobifunctional crosslinkers (bismaleimide- and bis-haloacetyl-based crosslinkers) to incorporate intramolecular crosslinking between two cysteines at position 67 and 60/58. This was performed in an otherwise Cys-free protein context. However, we found that the reaction was prone to many side reactions. Specifically, we observed that various by-products such as dimers, monoreactions, double reactions can be formed without intramolecular crosslinking and also unmodified protein can still be present in the solution.

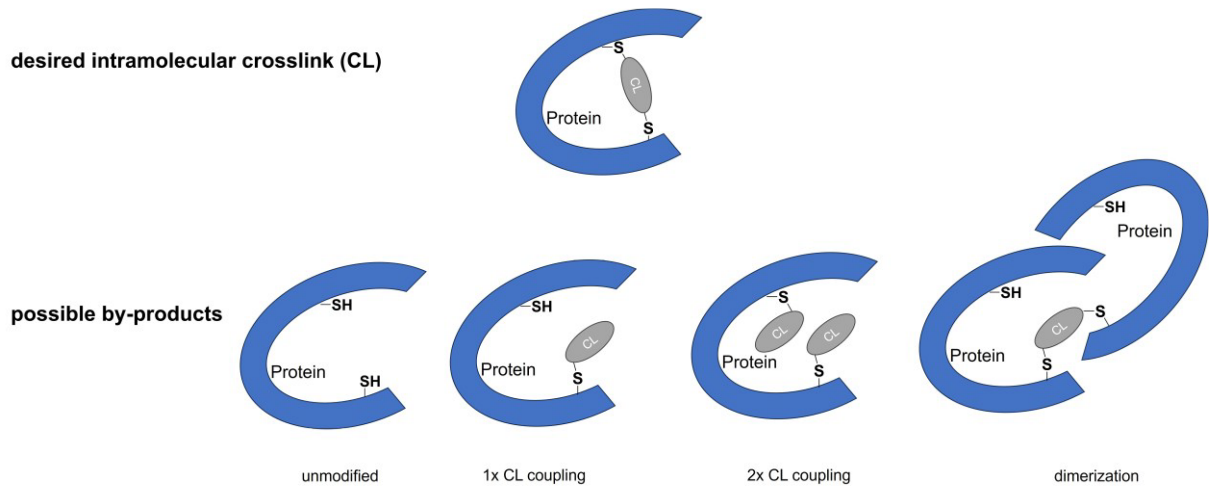

Figure 1: Summary of possible by-products in crosslinking with a homobifunctional crosslinker (CL).

Isolation of the desired intramolecularly bis-crosslinked products turned out to be exceedingly difficult. Since most by-products are very similar, separation by common chromatographic methods such as IEX or SEC was not impossible. After several purification steps, the samples still showed significant impurities, so we decided to discard this approach. We now mentioned this approach as well as our experiences in the manuscript by including: "We have also explored bis-electrophiles to incorporate intramolecular crosslinks between two cysteines at position 67 and 60/58. However, the reaction was prone to side reactions as various by-products were and isolation of the desired intramolecularly crosslinked product wasn't possible. Therefore, we focused total protein synthesis which allow a higher degree of reaction control."

Additional Questions:
Quality of experimental data, technical rigor: Top 5%

Significance to chemistry researchers in this and related fields: Top 5%

Broad interest to other researchers: Top 5%

Novelty: Top 5%

Is this research study suitable for media coverage or a First Reactions (a News & Views piece in the journal)?: Yes

Reviewer: 2

Recommendation: Reconsider after major revisions noted.

Comments:
The submitted manuscript "Automated flow peptide synthesis enables engineering of proteins with stabilized transient binding pockets" by Anna Charalampidou, Thomas Nehls, Christian Meyners, Satish Gandhesiri, Sebastian Pomplun, Bradley L. Pentelute, Frederik Lermyte and Felix Hausch demonstrates the use of chemical protein synthesis to produce conformationally locked derivatives of FKBP51. Furthermore, they show an improved affinity of conformationally specific ligands to these locked, synthetic derivatives. The manuscript is well written and provides an excellent example of the use of synthetic proteins in medicinal chemistry studies. However, there are some concerns regarding the analytical data that need to be addressed prior to publication of this study.

Major edits:

1.) The analytical data of the synthetic proteins (analytical HPLC and HR-LCMS) should be provided in both, the figure and the SI. In the SI it is currently missing, and in the manuscript figure the data is barely readable. Please enlarge analytical data in the manuscript and provide a full analytical data set in the SI.

We have improved the readability of Figure 2 (analytical HPLC and LC-MS) in the manuscript. The SI know also provides an enlargement of the corresponding full data set (SI3). Also, the order of the constructs has now been standardized.

2.) The authors claim that all samples have high purity, however, the 3rd derivative (and to some degree also the 5th) in Figure 2 show some larger impurities. Please comment on these.

We have changed the text to more accurately state that the samples all show sufficient purity. As the reviewer has noticed, the samples contain some shorter peptide chains (around 5000 Da); however, these do not interfere with the experiments presented in our manuscript. We have added MS data from the SEC-MS measurements (SI 6&7) and the ion mobility (SI 8) experiments to the SI to unambiguously show the sufficient purity of the synthesized samples.

Minor edits:

3.) In the abstract, the sentence "prepare proteins in a specific conformation" requires modification. The proteins are prepared with non-canonical amino acids enforcing this specific conformation, but the conformation is not prepared itself.

Sentence was changed to: "…generate proteins in a specific conformation…"

4.) In the abstract: I think "solid phase late-stage modifications" should be " solid-phase latestage modifications" (as in solid-phase peptide synthesis)

Sentence was changed to “…solid-phase late-stage modifications”

5.) in previous reports, AFPS has been used as an abbreviation for "automated fast-flow peptide synthesis" and here it is "automated flow peptide synthesis". For consistency, I would recommend to insert the "fast" to keep the original meaning.

In the manuscript the method is now referred to as “rapid automated flow peptide synthesis”

6.) For ion mobility MS (Figure 3C): please also show the "unfolded" control

The “unfolded” control is shown in the supporting information as figure SI 9. We mentioned this now in the caption of figure 3C.

7.) Supporting information: resin loading is missing in the materials section

Loading was inserted

Additional Questions:
Quality of experimental data, technical rigor: Moderate

Significance to chemistry researchers in this and related fields: High

Broad interest to other researchers: High

Novelty: High

Is this research study suitable for media coverage or a First Reactions (a News & Views piece in the journal)?: No

oc-2023-01283m.R2

Name: Peer Review Information for "Automated flow peptide synthesis enables engineering of proteins with stabilized transient binding pockets"

Second Round of Reviewer Comments

Reviewer: 2

Comments to the Author

The submitted manuscript "Automated flow peptide synthesis enables engineering of

proteins with stabilized transient binding pockets" by Anna Charalampidou, Thomas Nehls, Christian Meyners, Satish Gandhesiri, Sebastian Pomplun, Bradley L. Pentelute, Frederik Lermyte and Felix Hausch demonstrates the use of chemical protein synthesis to produce conformationally locked derivatives of FKBP51. Furthermore, they show an improved affinity of conformationally specific ligands to these locked, synthetic derivatives. The manuscript is well written and provides an excellent example of the use of synthetic proteins in medicinal chemistry studies. This reviewer therefore recommends publication after addressing the following comments:

- During the first round of revision, this reviewer asked for proper analytical analysis, and additional data was now provided by the authors. The authors claim that "the samples contain some shorter peptide chains (around 5000 Da); however, these do not interfere with the experiments presented in our manuscript." As opposed to this statement, the analytical data in the SI (page 16) unfortunately shows that the protein samples contain various impurities in the MS area of +/-250 Da. An additional SEC purification was carried out after folding of the protein, and the authors provide additional MS data demonstrating the significantly improved purity of the synthetic proteins. Please adjust the text and figures in the manuscript to accurately describe the purity of the protein samples.

- In their revised manuscript the authors write "However, the reaction was prone to side reactions as various by-products were formed and isolation of the desired intramolecularly crosslinked product wasn’t possible." Are these side-products or by-products?

Reviewer: 1

Comments to the Author

The authors have addressed all points I had raised and I can recommend to publish the work.

Author's Response to Peer Review Comments:

Dear Dr. Editor,

we revised the manuscript to according to the minor pending issues from Reviewer 2, as summarized in the revision summary. We also adressed the editorial comments.

Thank oyu very much for processing our manuscript,

Felix Hausch

Revision summary 2 for manuscript oc-2023-01283m

We addressed the reviewer comments as follows:

Reviewer(s)' Comments to Author:

Reviewer: 2

Recommendation: Publish in ACS Central Science after minor revisions noted.

Comments:

The submitted manuscript "Automated flow peptide synthesis enables engineering of proteins with stabilized transient binding pockets" by Anna Charalampidou, Thomas Nehls, Christian Meyners, Satish Gandhesiri, Sebastian Pomplun, Bradley L. Pentelute, Frederik Lermyte and Felix Hausch demonstrates the use of chemical protein synthesis to produce conformationally locked derivatives of FKBP51. Furthermore, they show an improved affinity of conformationally specific ligands to these locked, synthetic derivatives. The manuscript is well written and provides an excellent example of the use of synthetic proteins in medicinal chemistry studies. This reviewer therefore recommends publication after addressing the following comments:

- During the first round of revision, this reviewer asked for proper analytical analysis, and additional data was now provided by the authors. The authors claim that "the samples contain some shorter peptide chains (around 5000 Da); however, these do not interfere with the experiments presented in our manuscript." As opposed to this statement, the analytical data in the SI (page 16) unfortunately shows that the protein samples contain various impurities in the MS area of +/-250 Da. An additional SEC purification was carried out after folding of the protein, and the authors provide additional MS data demonstrating the significantly improved purity of the synthetic proteins. Please adjust the text and figures in the manuscript to accurately describe the purity of the protein samples.

We commented now on the impurities in the main text and highlighted the improvement in purity after additional chromatography.

- In their revised manuscript the authors write "However, the reaction was prone to side reactions as various by-products were formed and isolation of the desired intramolecularly crosslinked product wasn’t possible." Are these side-products or by-products?

If side-products are referred to as products of a reaction that are formed by competitive reactions, then in this case side-product is a more appropriate term than by-products, which are undesirable but nevertheless unavoidable products of a reaction. We changed byproducts to side-products in the mentioned sentence.

Additional Questions:

Quality of experimental data, technical rigor: High

Significance to chemistry researchers in this and related fields: Top 5%

Broad interest to other researchers: Top 5%

Novelty: High

Is this research study suitable for media coverage or a First Reactions (a News & Views piece in the journal)?: No

Reviewer: 1

Recommendation: Publish in ACS Central Science without change.

Comments:

The authors have addressed all points I had raised and I can recommend to publish the work.

Additional Questions:

Quality of experimental data, technical rigor: Top 5%

Significance to chemistry researchers in this and related fields: Top 5%

Broad interest to other researchers: Top 5%

Novelty: Top 5%

Is this research study suitable for media coverage or a First Reactions (a News & Views piece in the journal)?: Yes
